# The evolving landscape of the Warburg effect in gastric cancer: From molecular mechanisms to targeted therapy

**DOI:** 10.1002/ctm2.70772

**Published:** 2026-08-05

**Authors:** Guanmo Liu, Pan Li, Zizhuo Li, Jie Li, Chenggang Zhang, Yihua Wang, Weiming Kang, Xin Ye

**Affiliations:** ^1^ Department of General Surgery Peking Union Medical College Hospital Chinese Academy of Medical Sciences & Peking Union Medical College Beijing China

**Keywords:** gastric cancer, glycolysis, metabolic heterogeneity, targeted therapy, Warburg effect

## Abstract

**Background:**

Gastric cancer (GC) remains a major health burden because of late‐stage diagnosis and resistance to systemic therapy.

**Main body:**

In GC, the Warburg effect is driven by interconnected hypoxia and oncogenic signalling, non‐coding RNA networks and transcriptional and epigenetic rewiring. These regulators converge on glucose transport and core glycolytic enzymes, including HK2, PFKFB3, PKM2 and LDHA. The resulting increase in aerobic glycolysis, pyruvate‐to‐lactate conversion and lactate export supports biosynthesis, redox homeostasis and survival under metabolic stress. Beyond its bioenergetic role, lactate acts as a signalling metabolite that acidifies and remodels the tumour microenvironment. It impairs CD8‐positive T and natural killer cells function, promotes regulatory T cells accumulation and M2 macrophages polarisation, activates stromal cells and facilitates epithelial–mesenchymal transition and immune evasion. Together, tumour‐intrinsic metabolic adaptation and lactate‐mediated microenvironmental reprogramming contribute to resistance to chemotherapy, targeted therapy and immunotherapy. Translationally, metabolic imaging, circulating LDH and lactate‐related markers, exosomal glycolytic enzymes, gene‐expression signatures and lactylation‐related features may improve prognostic assessment, treatmentresponse prediction and patient stratification. These findings support biomarker‐guided combination strategies that target tumour metabolism, lactate production or transport and stromalimmune crosstalk alongside standard therapies. This review uniquely integrates direct GC‐derived evidence with indirect evidence from non‐GC models and clinical trials and grades biomarkers and metabolic interventions according to their translational maturity.

**Conclusion:**

Overall, the Warburg effect represents both a central driver of GC progression and a clinically relevant metabolic vulnerability.

**Key points:**

- The Warburg effect in gastric cancer is a multi‐module metabolic state, not a single glycolytic phenotype.
- Lactate links glycolytic reprogramming to stromal activation, immune evasion and therapy resistance.
- Microbiota, especially *H. pylori*, modulates glycolysis and creates actionable upstream vulnerabilities.
- Biomarker‐guided combinations, rather than isolated glycolytic inhibition, define the translational path forward.

## Graphical abstract

Hypoxia, oncogenic signaling, transcriptional and epigenetic remodeling, and *H. pylori*–microbiota interactions drive the Warburg effect in gastric cancer. Enhanced glycolysis and lactate export support biosynthesis, remodel the tumor microenvironment, promote stromal activation and immune suppression, and reveal actionable biomarkers and therapeutic opportunities targeting glycolysis and lactate metabolism.

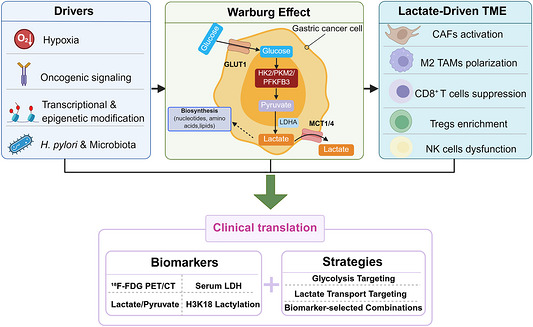

## 1 INTRODUCTION

Gastric cancer (GC) ranks among the top five malignancies in global incidence and mortality.^1^, ^2^ Despite advances in systemic therapy, a large proportion of patients are diagnosed at locally advanced or metastatic stages, and 5‐year overall survival (OS) drops sharply from 85.07% for stage I to 13.15% for stage IV.^3^ In resectable, locally advanced GC, gastrectomy is routinely paired with systemic therapy, most commonly perioperative FLOT (fluorouracil plus leucovorin, oxaliplatin and docetaxel) or fluoropyrimidine‐based adjuvant chemotherapy such as S‐1 or capecitabine–oxaliplatin after extended (D2) lymph‐node dissection, yet recurrence risk peaks within the first postoperative year.^4^, ^5^, ^6^, ^7^ In metastatic or unresectable GC, adding programmed cell death protein 1 (PD‐1) blockade to chemotherapy provides a survival benefit, but response is heterogeneous and primary resistance as well as acquired immune escape remains frequent.^8^, ^9^

The Warburg effect, also termed aerobic glycolysis, denotes preferential fermentation of glucose‐derived pyruvate to lactate even when oxygen is available and constitutes a hallmark bioenergetic phenotype in GC.^10^, ^11^, ^12^ Metabolomic profiling of metastatic GC models identified a shift towards enhanced glycolysis characterised by elevated lactate‐related signals and glucose depletion compared with non‐metastatic GC tumour models. A lactate‐enriched tumour microenvironment (TME) impairs CD8‐positive T cells function, promotes immune escape and reduces sensitivity to PD‐1 blockade.^13^, ^14^, ^15^ Consistently, serum lactate dehydrogenase (LDH)‐based indices and tumour glycolytic activity markers (e.g., ^1^ ^8^F‐FDG PET parameters, hexokinase 2 (HK2) expression) correlate with survival outcomes in GC patients treated with immune checkpoint inhibitors (ICIs).^16^, ^17^ Proteome‐scale lactylome profiling in GC has further identified thousands of lysine lactylation (Kla) sites, linking lactate accumulation to broad post‐translational remodelling.^18^, ^19^

Although recent reviews have summarised Warburg effect‐associated regulators and pivotal glycolytic enzymes in GC, most have focused on individual molecules or therapeutic targets rather than on how these findings converge into a clinically interpretable framework. In this review, we therefore propose a continuous mechanistic‐to‐translational axis that links tumour‐intrinsic genetic, transcriptional, epigenetic and epitranscriptomic regulation of glucose flux to metabolic heterogeneity across tumour, stromal and immune compartments, lactate‐driven TME remodelling, lactylation, microbiota‐associated metabolic modulation and biomarker‐guided therapeutic strategies. Importantly, we also distinguish direct GC‐derived evidence from indirect evidence obtained in other tumour types, allowing readers to assess the translational maturity of different biomarkers and metabolic interventions. By integrating these dimensions, this review aims to move beyond a catalogue of glycolytic enzymes and provide a framework for patient stratification, therapeutic prioritisation and rational combination strategies targeting the Warburg effect in GC. This evidence‐aware structure is intended to clarify which findings are close to clinical application and which remain hypothesis‐generating.

## 2 MECHANISMS AND REGULATION OF THE WARBURG EFFECT

### 2.1 The role of the Warburg effect in tumours

The Warburg effect reflects coordinated metabolic rewiring in which cancer cells sustain elevated glucose uptake and glycolysis despite adequate oxygen, coupled with preferential pyruvate‐to‐lactate conversion and lactate export. This program integrates oncogenic and tumour‐suppressor signalling with biosynthetic shunts and metabolite signalling, supporting proliferation, redox balance, epigenetic plasticity and immune evasion.^20^, ^21^

Cancer cells elevate glucose transporter 1 (GLUT1)‐dependent glucose import and commit intracellular glucose to glycolysis through HK2‐catalysed phosphorylation to glucose‐6‐phosphate.^22^ Growth factor stimulation induces co‐endocytosis of receptor tyrosine kinases and GLUT1, forming signalling‐enriched vesicles that deliver glucose near mitochondria‐associated glycolytic enzymes.^23^ GLUT1 availability can be rate‐limiting for glycolysis, as demonstrated in chimeric antigen receptor T cells where enforced GLUT1 expression enhanced glycolytic capacity.^24^ A glucose‐responsive CRL4^COP1^–p53 degradation axis further reinforces glycolytic programs under glucose‐replete conditions.^25^

To avoid overextension, the non‐GC examples in this mechanistic overview are presented as referential data for conserved metabolic principles and should not be interpreted as direct validation in GC unless GC‐derived evidence is explicitly cited. 6‐phosphofructo‐2‐kinase/fructose‐2, 6‐bisphosphatase‐3 (PFKFB3) produces fructose‐2,6‐bisphosphate, a potent allosteric activator of phosphofructokinase‐1 (PFK1), strengthening the commitment step of glycolysis.^26^, ^27^, ^28^ PFKFB3‐driven glycolytic activation has also been validated in non‐GC tumour models. In diffuse midline glioma, extracellular‐signal‐related kinase 5 (ERK5) promotes glycolysis through myocyte enhancer factor 2A (MEF2A)‐dependent PFKFB3 induction, whereas genetic or pharmacologic PFKFB3 inhibition suppresses proliferation and tumour growth. In hepatocellular carcinoma, PFKFB3 blockade reduces tumour growth and promotes vascular normalisation, linking glycolytic inhibition to improved tumour vascular function.^29^, ^30^ Pyruvate kinase M2 (PKM2) functions as both a metabolic enzyme and a signalling integrator. Growth factor–ERK signalling phosphorylates PKM2 at serine775 (Ser37), promoting its low‐activity dimeric state and nuclear import, where phosphoenolpyruvate serves as a phosphate donor for histone H3 threonine11 phosphorylation and hypoxia‐inducible factor‐1α (HIF‐1α)‐driven transcription of glycolytic genes.^31^, ^32^, ^33^ Structural perturbations—including H_2_S‐induced S‐sulfhydration at cysteine326 and stress‐induced aggregation—dynamically tune PKM2 between enzymatic and signalling functions in tumours.^34^, ^35^, ^36^ The pyruvate dehydrogenase complex (PDC) controls pyruvate entry into the tricarboxylic acid (TCA) cycle and inhibition of the PDC can drive aerobic glycolysis and lactate release.^37^, ^38^ In lung cancer, polo‐like kinase 1 phosphorylated the pyruvate dehydrogenase E1‐α subunit, destabilising the PDC, suppressing oxidative phosphorylation and shifting metabolism towards increased glycolysis.^39^

LDH regenerates cytosolic NAD^+^ to sustain glycolytic throughput. Systemic LDH inhibition in mouse tumours redirected intratumoural glucose uptake towards T cells and enhanced ICIs efficacy.^10^, ^40^, ^41^ Monocarboxylate transporters export lactate with protons, buffering intracellular pH and sustaining high glycolytic flux.^42^, ^43^ In a first‐in‐human trial, the monocarboxylate transporter 1 (MCT1) inhibitor AZD3965 achieved target engagement but limited anti‐tumour activity; pharmacologic monocarboxylate transporter 4 (MCT4) blockades combined with anti‐PD‐L1 raised intratumoural pH, enhanced T cells activation and prolonged survival, underscoring restrained lactate efflux as a key tumour‐intrinsic constraint on anti‐tumour immunity.^44^, ^45^

Glycolytic intermediates are diverted to the pentose phosphate pathway (PPP), serine‐one‐carbon metabolism and acetyl coenzyme A (acetyl‐CoA)‐dependent histone acetylation. Parallel anabolic pathways siphon glycolytic intermediates to support biosynthesis and redox homeostasis. In pancreatic ductal adenocarcinoma, a GLUT1/aldolase B (ALDOB)/glucose‐6‐phosphate dehydrogenase (G6PD) axis activated the oxidative PPP and remodelled glucose metabolism, resulting in chemoresistance in patient‐derived organoids and organoid‐derived xenografts. Pharmacologic inhibition of GLUT1/G6PD or genetic interventions (such as GLUT1 knockdown or ALDOB overexpression) reversed these metabolic features and sensitised tumours to gemcitabine and 5‐fluorouracil (5‐FU) in vitro and in vivo.^46^ At the chromatin end of carbon flux, oncogenic PI3K–AKT signalling activates ATP‐citrate lyase (ACLY) via phosphorylation at Ser455, increasing the pool of nucleo‐cytosolic acetyl‐CoA derived from mitochondrial citrate export. The enriched acetyl‐CoA subsequently fuels p300‐dependent histone H3K27 acetylation at the PD‐L1 promoter and up‐regulates PD‐L1 to promote tumour immune evasion. Conversely, ACLY inhibition reinvigorates cytotoxic T cells and enhances immune checkpoint blockade efficacy.^47^, ^48^ This places glycolysis‐fed citrate export and histone acetylation squarely within the broader Warburg network that drives epigenetic regulation of immune escape.

In GC, this conserved glycolytic program acquires disease‐specific features that provide the rationale for the following sections. First, GC cells integrate hypoxia, PI3K/AKT/mTOR, c‐Myc, Wnt/beta‐catenin, Hippo/YAP and non‐coding RNA (ncRNA) circuits to stabilise HK2‐, PFKFB3‐, PKM2‐ and LDHA‐centred glycolysis. Second, the gastric TME actively participates in this program: GC mesenchymal stem cells (GC‐MSCs), cancer‐associated fibroblasts (CAFs), macrophages and T cells exchange cytokines, exosomes and lactate, thereby coupling tumour‐intrinsic glycolysis to immune evasion and chemoresistance. Third, the stomach‐specific microbial context, including *H. pylori* and selected microbial metabolites, adds an additional regulatory layer to glycolytic remodelling. These GC‐specific features link the general principles of aerobic glycolysis to the detailed molecular pathways, metabolic heterogeneity, microenvironmental remodelling and translational strategies discussed below.

### 2.2 Critical signalling pathways of the Warburg effect in GC

**FIGURE 1:**
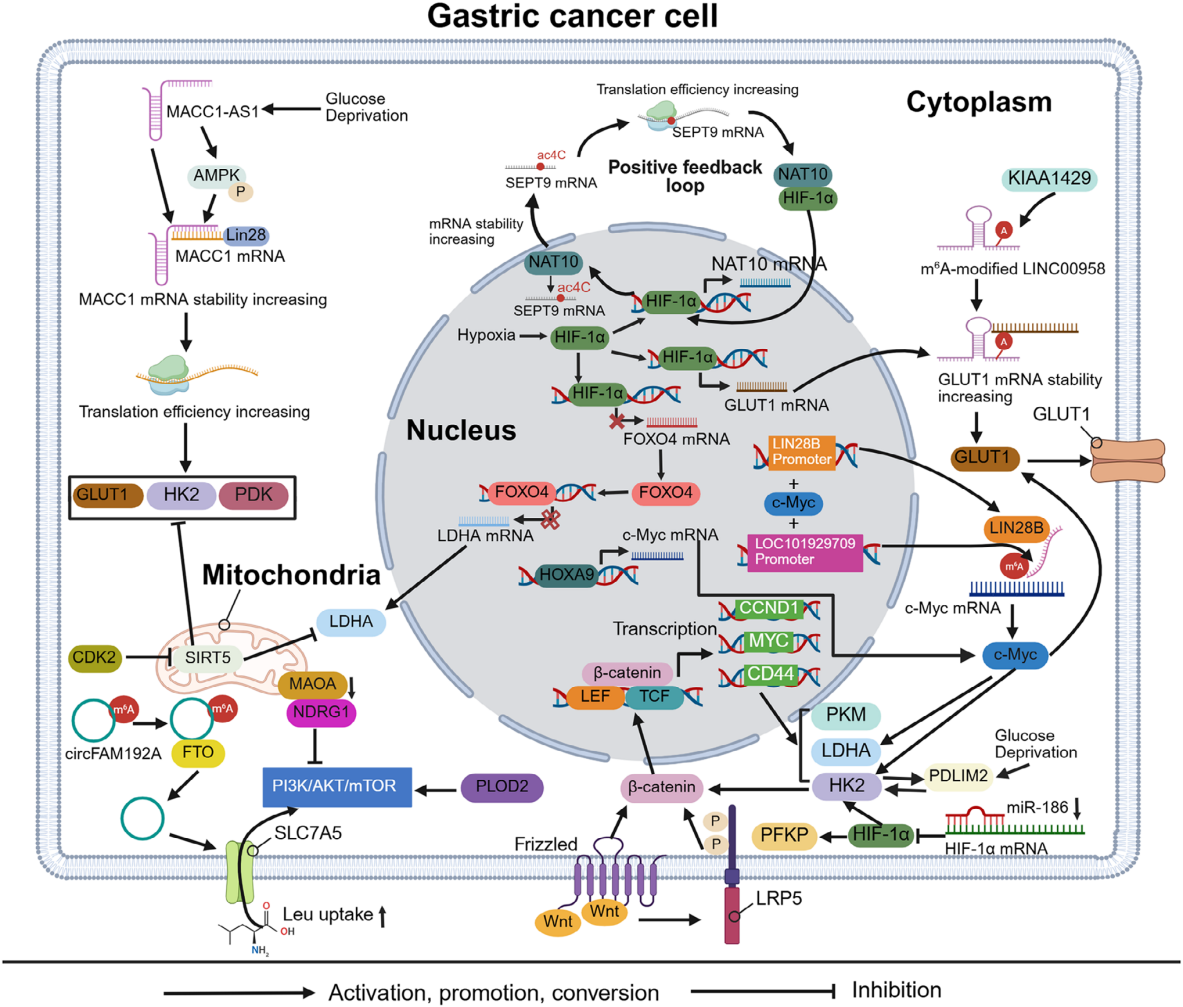
The molecular mechanisms and signalling networks promoting the Warburg effect in gastric cancer cells. This schematic summarises how metabolic stress, RNA modifications, transcription factors, non‐coding RNAs and oncogenic signalling pathways converge to regulate core glycolysis‐associated effectors, thereby driving glycolytic reprogramming and metabolic adaptation in gastric cancer.

The Warburg effect is a key metabolic reprogramming event in GC. Proteomic profiling of non‐cardia GC reveals reduced abundance of multiple enzymes in the citrate cycle and oxidative phosphorylation pathways, together with altered glycolytic enzyme expression, such as glycolytic enzyme enolase 1 (ENO1), indicating a shift in energy production from mitochondrial respiration towards a Warburg‐type glycolytic phenotype in tumour tissue compared with matched non‐neoplastic gastric mucosa.^49^ Integrative multi‐omics analyses demonstrate the concept that GC develop a pronounced dependence on aerobic glycolysis, marked by up‐regulation of glycolytic enzymes, glucose transporters and LDH isoforms, which is orchestrated by interconnected oncogenic signalling hubs.^12^, ^50^, ^51^, ^52^ HIF‐1α, PI3K/AKT/mTOR and c‐Myc‐centred epitranscriptomic programs cooperate with Wnt/β‐catenin and Hippo/YAP pathways, as well as stromal signals from GC‐MSCs. These networks converge on key glycolytic effectors, including HK2, PKM, LDHA and glucose transporters, sustaining the Warburg effect in GC (Figure 1).^12^, ^53^, ^54^, ^55^, ^56^, ^57^, ^58^

Under hypoxia, HIF‐1α up‐regulates GLUT1 and glycolytic enzymes in GC, promoting resistance to chemotherapy and radiotherapy.^59^, ^60^, ^61^ The study by Wang et al. showed that HIF‐1α bound to hypoxia‐response elements within the forkhead box O4 (FOXO4) promoter and repressed FOXO4 transcription in hypoxia‐exposed GC. FOXO4 directly suppressed LDHA transcription. Furthermore, FOXO4 overexpression reduced glycolytic flux and suppressed GC progression in xenograft models.^62^ The study by Liu et al. indicated that microRNA (miRNA)‐186 directly targeted HIF‐1α and down‐regulated HK2 and platelet‐type phosphofructokinase (PFKP), suppressing aerobic glycolysis and restraining GC cell proliferation.^63^ Furthermore, a recent study identified that HIF‐1α induces RNA acetyltransferase N‐acetyltransferase 10 (NAT10)‐mediated N4‐acetylcytidine (ac4C) modification of SEPT9 messenger RNA (mRNA) under hypoxia, establishing a NAT10/SEPT9/HIF‐1α‐positive feedback loop that drives glycolysis addiction and intrinsic resistance to anti‐angiogenic therapy.^64^ Hypoxia‐inducible 5′‐Nucleotidase Ecto (NT5E/CD73) is up‐regulated under hypoxia and promotes the Warburg effect in GC via extracellular adenosine accumulation. These metabolic changes support subcutaneous xenograft growth. Conversely, genetic knockdown of CD73 or pharmacologic inhibition of its ectonucleotidase activity markedly attenuates tumour glycolysis and dampens the Warburg effect.^65^

Canonical Wnt/β‐catenin signalling provides an additional mechanism regulating glycolytic reprogramming in GC and can be activated by overexpression of low‐density lipoprotein receptor‐related protein 5 (LRP5).^66^, ^67^ LRP5, a co‐receptor for Wnt ligands, is markedly up‐regulated in GC tissues and associated with advanced stage and poor survival.^67^, ^68^ Acting as a cytoskeletal adaptor, PDZ and LIM domain protein 1 (PDLIM1) physically associates with HK2 to form an HK2–PDLIM1 complex. Through the PDLIM1–HK2 complex, Wnt/β‐catenin signalling is activated particularly under glucose‐deprived conditions, thereby amplifying the Warburg effect and promoting proliferation, migration, invasion and apoptosis resistance in GC.^56^, ^69^ In sum, Wnt co‐receptors and cytoskeletal adaptor proteins function as critical upstream regulators that modulate HK2‐dependent glycolysis and couple cytoskeletal remodelling to Wnt/β‐catenin‐mediated transcriptional programs, enhancing the Warburg effect in GC.^56^, ^70^

Beyond classic metabolic enzymes, the intricate crosstalk between cell‐cycle machinery and oncogenic transcriptional networks constitutes a critical layer of control over the Warburg effect, tightly coupling cell proliferation with glycolytic reprogramming in GC. Cyclin‐dependent kinase 2 (CDK2) suppresses tumour‐suppressive sirtuin 5 (SIRT5) to foster aerobic glycolysis, whereas genetic silencing of CDK2 up‐regulates SIRT5, decreases extracellular acidification rates and the expression of key glycolytic genes (GLUT1, HK2, LDHA and pyruvate dehydrogenase kinase (PDK)) and enhances mitochondrial respiration. SIRT5 re‐expression inhibits glycolysis and tumour growth in vivo.^71^ Under glucose deprivation, AMPK‐dependent up‐regulation of metastasis‐associated in colon cancer 1 (MACC1) enhances HK2, PDK and LDHA expression and activities, which collectively supports cell survival and relieves apoptosis in metabolically stressed cells and xenograft tumours. High MACC1 expression and activation of the MACC1‐AS1 (cognate antisense long ncRNA (lncRNA) of MACC1)/MACC1 axis are associated with advanced clinicopathological stage and poor prognosis in GC. Functionally, this axis promotes cell‐cycle progression by alleviating glucose‐deprivation‐induced S‐phase arrest, supporting MACC1 as a glycolysis‐coupled oncogenic driver that links metabolic reprogramming to cell‐cycle control.^72^, ^73^ In addition to MACC1, Spalt like transcription factor 4 (SALL4), a critical driver of cell‐cycle progression, directly binds to the HK2 promoter to transactivate its expression in GC cells. This SALL4–HK2 axis improves glycolytic flux and genetic silencing of HK2 abolishes SALL4‐driven glycolytic reprogramming, thereby reducing SALL4‐induced GC cell proliferation, migration and invasion.^74^, ^75^ Consistent with the notion that proliferative programs are tightly wired into glycolytic machinery, centromere protein U (CENPU), a kinetochore‐associated regulator of mitosis, functionally links cell‐cycle control to tumour metabolism by up‐regulating high‐mobility group box 2 (HMGB2), which in turn enhances glycolysis and proliferation in GC cells. In AGS cells, CENPU silencing decreases HMGB2 expression and significantly reduces glucose consumption and lactate production, indicating that CENPU promotes glycolytic reprogramming in an HMGB2‐dependent manner.^76^, ^77^, ^78^

Monoamine oxidase A (MAOA), classically recognised as a mitochondrial enzyme responsible for monoamine neurotransmitter catabolism, is significantly down‐regulated in GC tissues. Its ectopic overexpression suppresses tumour growth and aerobic glycolysis by physically interacting with N‐myc downstream‐regulated gene 1 protein (NDRG1) and dampening activation of PI3K/AKT/mTOR signalling cascade. In MAOA‐overexpressing GC cells, pharmacological inhibition with the selective MAOA inhibitor clorgyline abolished these tumour‐suppressive and anti‐glycolytic effects.^79^, ^80^ Procollagen‐lysine,2‐oxoglutarate 5‐dioxygenase 2 (PLOD2) has been identified as a glycometabolism‐related oncogene in GC. Ye et al. showed that PLOD2 promotes GC progression through coordinated extracellular matrix remodelling, PI3K/AKT/mTOR‐driven glycolytic reprogramming and the formation of an immunosuppressive TME enriched in M2 macrophages. Consistently, elevated PLOD2 expression was associated with immune evasion and poor clinical prognosis in GC.^81^ The leucine transporter solute carrier family 7 member 5 (SLC7A5) is up‐regulated in oxaliplatin‐resistant GC cells, and its knockdown reduces glycolytic flux, subsequently re‐sensitising tumours to oxaliplatin both in vitro and in xenograft models.^82^ Mechanistically, complementary studies in GC showed that SLC7A5‐dependent leucine uptake activated mTOR signalling downstream of fat mass and obesity‐associated protein (FTO)‐circFAM192A, and that FTO also amplified PI3K/AKT signalling, positioning SLC7A5 within the broader PI3K/AKT/mTOR network that linked amino acid transport to glycolysis‐driven survival and chemoresistance.^83^, ^84^ Moreover, Lu et al. suggested that the pharmacological blockade of PI3K with LY294002 in GC mitigated the Warburg effect by lowering the expression of phosphorylated AKT and mTOR as well as PKM2, hence reducing LDH activity and lactate outputs.^85^

In addition to transcriptional regulation, an extensive network involving epitranscriptomic modifications and lncRNAs forms another crucial layer of regulation. By modulating processes such as mRNA stability and alternative splicing, often in conjunction with key oncogenes like c‐Myc, these circuits finely tune glycolytic enzyme expression and metabolic flux. As a member of the HOX gene family, HOXA9 directly binds the c‐Myc promoter, up‐regulating glycolytic enzymes.^86^ LncRNA LINC00958 is methylated and stabilised by the N6‐methyladenosine (m6A) methyltransferase KIAA1429 in GC cells. The m6A‐modified LINC00958 associates with GLUT1 mRNA and, in an m6A‐dependent manner, reinforces GLUT1 mRNA stability, thus elevating GLUT1 expression and strengthening aerobic glycolysis.^87^, ^88^ The m6A reader insulin‐like growth factor 2 mRNA‐binding protein 1 (IGF2BP1) is up‐regulated in GC and serves as an adverse prognostic factor. IGF2BP1 recognises m6A‐modified c‐Myc transcripts and stabilises c‐Myc mRNA, consequently augmenting c‐Myc expression and its glycolysis‐related transcriptional program.^58^ Moreover, the lncRNA LOC101929709 facilitates the interaction between the RNA‐binding protein LIN28B and c‐Myc mRNA, further intensifying the Warburg effect in GC.^89^ Beyond these m6A‐centred metabolic circuits, the cancer‐associated lncRNA colon cancer‐associated transcript 1 (CCAT1) directly binds to polypyrimidine tract‐binding protein 1 (PTBP1) and stabilises it by limiting ubiquitin‐proteasome‐mediated degradation. Stabilised PTBP1 functions as a key splicing regulator of PKM pre‐mRNA, shifting isoform expression from PKM1 towards PKM2 and increasing the PKM2/PKM1 ratio to amplify aerobic glycolytic flux.^90^ These findings indicate that m6A modifications and lncRNA networks, often converging on key hubs like c‐Myc, integrate multiple signals to regulate mRNA processing and stability, thereby consolidating the Warburg effect in GC.

In GC, the Hippo/YAP1 signalling axis, together with its scaffold proteins, acts as a crucial signalling hub that couples metabolic reprogramming with tumour‐microenvironmental crosstalk to drive cancer cell survival and chemoresistance.^91^, ^92^ The LIM‐domain scaffold protein Ajuba is markedly up‐regulated and its overexpression promotes tumour cell proliferation and enhances cisplatin resistance while concomitantly increasing GLUT1 expression and glucose uptake in GC. Ajuba attenuates Hippo pathway activity and elevates YAP protein levels, thereby sustaining YAP‐dependent induction of anti‐apoptotic and metabolic effectors such as Bcl‐xL and GLUT1, functionally coupling Hippo pathway inhibition to augmented glycolytic metabolism and pro‐survival signalling in GC cells.^93^ Overexpression of YAP1 in 5‐FU‐resistant GC cells promotes the secretion of IL‐3. IL‐3 skews tumour‐associated macrophages (TAMs) towards an M2 phenotype and induces GLUT3‐dependent glycolytic reprogramming in these macrophages. The reprogrammed TAMs subsequently secrete CCL8, which activates the Janus kinase 1/signal transducer and activator of transcription 3 (JAK1/STAT3) signalling pathway and reinforces 5‐FU resistance.^94^ These findings suggest that Hippo/YAP signalling, orchestrated by scaffold proteins like Ajuba and reciprocal cytokine crosstalk within the microenvironment, coordinates glycolysis in both cancer cells and stromal compartments to reinforce the Warburg effect and treatment resistance.

Furthermore, GC‐MSCs amplify the Warburg effect through multiple secreted factors. In GC‐MSCs, up‐regulated G6PD activates NF‐κB signalling and boosts the secretion of hepatocyte growth factor (HGF). GC‐MSCs‐derived HGF, through a c‐Myc‐HK2 signal axis in GC, amplifies glycolytic flux.^95^ GC‐MSCs secrete IL‐8, which signals through CXC chemokine receptor 1 (CXCR1) and CXC chemokine receptor 2 (CXCR2), particularly CXCR2, on GC cells. This IL‐8–CXCR2 engagement triggers AKT‐dependent phosphorylation and nuclear translocation of HK2. Once translocated, phosphorylated HK2 cooperates with HIF‐1α to promote PD‐L1 transcription. The resulting enhancement of glycolytic flux drives excessive lactate accumulation and blunts CD8^+^ T cell effector function.^57^, ^96^ GC‐MSCs‐derived TNF‐α induces the expression of B7 homolog 3 (B7H3) on adjacent GC cells. B7H3 up‐regulation is associated with enhanced glycolytic activity and reduced sensitivity to oxaliplatin and paclitaxel. In this GC‐MSCs‐driven context, silencing B7H3 or pharmacologically restraining glycolysis with 2‐deoxy‐D‐glucose (2‐DG) markedly attenuates oxaliplatin‐ and paclitaxel‐resistance in GC cells.^97^ These stromal and immune signalling circuits and lactate‐mediated suppression of effector immune cells underscore the Warburg effect as a crucial metabolic axis in the GC TME and help explain how metabolic remodelling is intertwined with immune escape and resistance to both chemotherapy and ICIs.

### 2.3 Transcriptional and epigenetic regulation of the Warburg effect in GC

The Warburg effect in GC is governed by a multifaceted network of transcriptional and epigenetic reprogramming. Massive ncRNA networks heavily modulate key glycolytic enzymes.^12^, ^98^, ^99^, ^100^, ^101^ Concurrently, epigenetic reprogramming reinforces this hyper‐glycolytic state: lactate‐induced histone lactylation drives oncogene transcription and immunosuppression, while DNA hypermethylation silences opposing gluconeogenic pathways.^11^, ^101^, ^102^, ^103^, ^104^, ^105^, ^106^, ^107^, ^108^ The coordinated networks of ncRNAs, histone modifications (particularly Kla) and DNA methylation act synergistically to help maintain this Warburg‐type glycolytic program, linking elevated glycolytic flux to GC progression, therapeutic resistance and dampened anti‐tumour immunity.

miRNAs and lncRNAs constitute a major layer of post‐transcriptional regulation that finely modulates key glycolytic enzymes and thus reprograms the Warburg effect in GC. miRNA‐181b suppresses HK2, reducing glucose uptake and lactate production while elevating intracellular ATP. Ultimately, this metabolic shift constrains aerobic glycolysis and suppresses cell proliferation.^109^ The long intergenic non‐protein coding RNA 242 (LINC00242) is up‐regulated in GC and serves as a competing endogenous RNA to sponge miRNA‐1‐3p, accordingly relieving repression of G6PD. In GC cells, silencing LINC00242 or G6PD, or overexpressing miRNA‐1‐3p, reduces glucose consumption and lactate production while inhibiting proliferation and promoting apoptosis. Collectively, these findings indicate that the LINC00242/miRNA‐1‐3p/G6PD axis supports the Warburg effect in GC.^110^ LncRNA H19 in GC up‐regulates LDHA and amplifies glycolytic flux via the H19/miRNA‐519d‐3p/LDHA axis, creating a lactate‐rich microenvironment that dampens anti‐tumour immune responses and facilitates immune escape.^111^ A complementary study illustrated that H19 also promoted aerobic glycolysis through a distinct H19/miRNA‐19a‐3p/phosphoglycerate kinase 1 (PGK1) pathway, further underscoring H19 as a key regulator of the Warburg effect in GC.^112^ Another lncRNA, HAGLR sponges miR‐338‐3p to release LDHA from repression, enhancing LDHA‐driven glycolysis and facilitating 5‐FU resistance.^113^ Furthermore, lncRNA‐ABHD11‐AS1, up‐regulated in GC, boosts aerobic glycolysis by increasing the expression of the transcription factor c‐Myc.^114^, ^115^

Circular RNAs (circRNAs), as highly stable ncRNAs with covalently closed loops, constitute another layer of the network regulating aerobic glycolysis. CircCUL3 acts as a competing endogenous RNA for miRNA‐515‐5p to sustain STAT3 expression. Subsequently, STAT3 binds to the HK2 promoter and drives HK2 transcription, leading to increased aerobic glycolysis in GC.^116^ CircRNA derived from ribosomal protein S19 (circRPS19), propels HK2‐mediated aerobic glycolysis through the miRNA‐125a‐5p/USP7 axis. CircRPS19, regarded as a molecular sponge for miRNA‐125a‐5p, enhances USP7 expression and USP7 deubiquitinates, which then deubiquitinates and stabilises HK2 protein, increasing glycolytic flux.^117^ CircATP2B1 sustains the Warburg effect in GC by sponging the miRNA‐326 gene cluster, hence weakening their repression of PKM2 and raising lactate production and ATP generation in cancer cells.^118^ Collectively, circRNAs form an additional regulatory tier that stabilises the Warburg effect in GC by sustaining glycolytic enzyme activity and lactate production.

Global lysine lactylome analysis identified 2375 Kla sites across 1014 proteins in GC. Combined with lactylomics and isotope‐tracing studies in gastrointestinal tumours, these findings support that lactate derived from the Warburg effect is reutilised as a metabolic donor to drive the lactylation of glycolytic enzymes and chromatin‐associated regulators.^119^, ^120^, ^121^ Histone H3 lysine 18 lactylation (H3K18la) is elevated in GC tissues and tightly linked to LDHA‐driven glycolytic flux. Lactate accumulation within the TME enriches H3K18la at the vascular cell adhesion molecule 1 (VCAM1) promoter, thereby boosting VCAM1 transcription. Elevated VCAM1 subsequently activates the AKT/mTOR signalling pathway, which in turn reinforces the Warburg effect in GC.^103^, ^122^, ^123^ H3K18la also accumulates at the PD‐L1 promoter, increasing PD‐L1 expression and triggering epithelial–mesenchymal transition (EMT) and immune evasion.^124^ In addition, in GC, SIRT1 functions as a H3K18 delactylase that restrains an oncogenic lncRNA H19‐glycolysis‐histone lactylation circuit. Loss of SIRT1 increases H3K18la at the H19 promoter, thereby up‐regulating H19 and amplifying LDHA‐dependent aerobic glycolysis and lactate production. The resulting lactate accumulation further enhances H3K18la, establishing a positive feedback loop that reinforces the Warburg effect.^112^, ^125^

Epigenetic silencing of gluconeogenic rate‐limiting enzymes, particularly FBP1 and FBP2, via promoter CpG island hypermethylation serves as a critical mechanism to sustain the Warburg effect in GC. This DNA‐methylation‐mediated repression creates a stable metabolic switch that promotes aerobic glycolysis.^98^, ^105^, ^126^ Recent data elucidated that DNA methyltransferase 3a (DNMT3a) bound to the FBP2 promoter and promoted its hypermethylation in GC, thereby muting an anti‐glycolytic gluconeogenic enzyme and aligning with poorer patient survival.^106^, ^107^

### 2.4 Regulation by intratumoural microbiota

Distinct from other gastrointestinal malignancies, GC unfolds in a stomach ecosystem first reshaped by *Helicobacter pylori* (*H. pylori*) driving chronic gastritis, gastric atrophy and subsequent hypochlorhydria, which weakens the acid barrier and permits expansion of non‐H. pylori taxa with lesion‐ and tumour‐enriched patterns. In this setting, tumour‐associated microbes may amplify the Warburg effect by increasing epithelial glucose uptake and glycolytic throughput, sustaining an acidic, lactate‐abundant TME and biasing infiltrating immune cells towards glycolysis, thereby prolonging inflammation and undermining anti‐tumour immunity (Table 1).^127^, ^128^, ^129^, ^130^

**TABLE 1:** Microbial modulation of the Warburg effect in GC: mechanisms and evidence levels.

Microbial factor	Target	Mechanism	Function on the Warburg effect or TME	Evidence source/study object	Evidence level	References
Microbiome‐derived butyrate	Wnt/β‐catenin/c‐Myc signalling axis	Down‐regulation of glycolytic enzymes and nuclearβ‐catenin translocation	Alleviates the Warburg effect to reduce tumour migration and invasion	Direct GC preclinical evidence, mainly GC cell and microenvironment‐related experimental models	Preclinical GC mechanistic evidence	^66^
*H. pylori* (urease)	TLR2	Induction of HIF‐1α; up‐regulation of GLUT1 and LDH‐5	Drives an early glycolytic shift in the gastric epithelium, which sustains bacterial persistence	Gastric epithelial infection models; *H. pylori*‐related gastric carcinogenesis context	Direct gastric epithelial/preclinical evidence	^131^
*H. pylori* (general infection)	Lonp1	HIF‐1α‐dependent induction of Lonp1	Sustains mitochondrial quality via mitochondrial unfolded protein response to increase the Warburg effect and supports infection‐associated epithelial overgrowth	*H. pylori*‐infected gastric epithelial cell models	Direct gastric epithelial/preclinical evidence	^133^
*H. pylori* (CagA)	PI3K/AKT signalling/SIRT3	Destabilisation of mitochondrial SIRT3; increased ROS generation; stabilisation of HIF‐1α; activation of the PI3K/AKT pathway	Induces metabolic reprogramming towards the Warburg effect and confers 5‐FU chemoresistance	GC cell models and *H. pylori* virulence‐factor studies	Direct GC preclinical evidence	^134^, ^135^
*Akkermansia muciniphila* (PEA)	FUBP1	Inhibition of FUBP1‐mediated transcriptional activity through direct PEA binding	Attenuates the Warburg effect and sensitises GC to oxaliplatin	Direct GC preclinical models involving microbial metabolite intervention	Preclinical GC mechanistic and therapeutic evidence	^141^

*H. pylori* infection imposes an early glycolytic bias in the gastric epithelium, largely orchestrated by the HIF‐1α and multiple bacterial virulence factors. First, the established colonisation factor, urease, acts as a non‐enzymatic trigger for Toll‐like receptor‐2 (TLR2) signalling to induce HIF‐1α expression. Once activated, HIF‐1α coordinately up‐regulates key metabolic players such as GLUT1,^12^, ^60^, ^131^, ^132^ and the mitochondrial Lon protease 1 (Lonp1) in *H. pylori*‐infected gastric epithelial cells.^133^ Together, these factors promote the Warburg effect, fuelling infection‐associated epithelial overgrowth. *H. pylori* cytotoxin‐associated gene A (CagA) dampens 5‐FU responsiveness in GC cells by skewing metabolism towards a Warburg‐like glycolytic state. Pharmacologic inhibition of AKT signalling or glycolysis partly restores 5‐FU cytotoxicity. Separately, CagA has been shown to destabilise mitochondrial SIRT3, increasing reactive oxygen species (ROS) and augmenting HIF‐1α activity, which up‐regulates HIF‐1α‐dependent glycolysis‐related programs in GC models.^134^, ^135^

Progressive gastric gland atrophy with hypochlorhydria disrupts the gastric ecological barrier, facilitating colonisation by oropharyngeal and enteric lineages.^128^, ^136^, ^137^ Community‐scale profiling of the gastric microbiota in GC repeatedly delineates discrete community states, often stratified by *H. pylori* dominance, across the carcinogenic sequence and within tumours. These non‐uniform configurations vary with clinicopathological context and intersect with Warburg‐like metabolic reprogramming.^138^, ^139^, ^140^ In GC, *Akkermansia muciniphila*‐derived pentadecanoic acid (PEA) suppresses the Warburg effect and improves oxaliplatin responsiveness. Mechanistically, far upstream element‐binding protein 1 (FUBP1) sustains the Warburg effect in GC cells, whereas PEA antagonises FUBP1 and curtails glycolytic flux, thereby restoring oxaliplatin responsiveness.^141^ This metabolic control is immunologically consequential because lactate accumulation in GC tumour‐infiltrating T cells associates with diminished T helper 1 cells and cytotoxic T lymphocyte compartments, and lactate can blunt nuclear factor of activated T cells and IFN‐γ production in T and natural killer (NK) cells, favouring immune escape.^142^, ^143^ Furthermore, short‐chain fatty acid butyrate from microbiome attenuated migration and invasion and dampened aerobic glycolysis in GC, accompanied by reduced expression of GLUT1, HK2 and LDHA, and by suppression of Wnt/β‐catenin/c‐Myc signalling. Butyrate also lowered PD‐L1 and IL‐10 in CD68‐positive macrophages in GC, implying that selected microbial metabolites can reshape both glycolytic wiring and immunosuppressive tone within the TME.^66^, ^144^ These mechanistic findings also suggest that *H. pylori* is not only a microbial regulator of glycolytic remodelling but also a clinically actionable upstream target, as discussed in the therapeutic translation section.

## 3 METABOLIC HETEROGENEITY OF THE WARBURG EFFECT IN GC

The Warburg effect in GC is not a uniform metabolic program but a heterogeneous landscape shaped by the aberrant expression of key glycolytic enzymes, the moonlighting functions of metabolites and the spatiotemporal remodelling of the TME. Understanding this heterogeneity provides the foundation for precise metabolic interventions.

### 3.1 Glycolytic enzyme plasticity and multifunctionality

**FIGURE 2:**
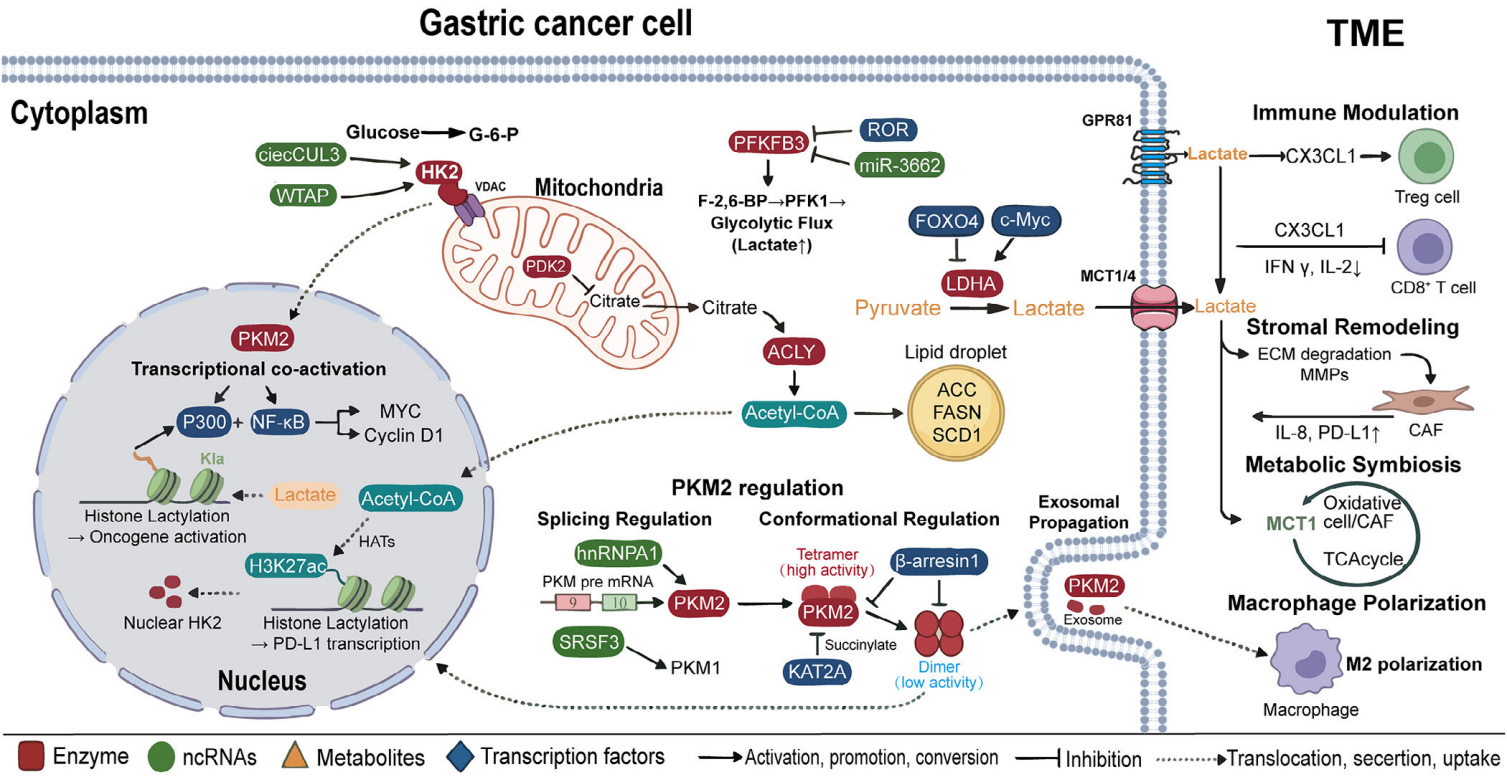
Coordinated regulation of glycolytic enzymes and metabolite signalling in the Warburg effect of gastric cancer. This schematic depicts the multilayered control of HK2, PFKFB3, PKM2 and LDHA in GC. HK2 is stabilised by post‐transcriptional and post‐translational modifications, anchoring to mitochondria to couple glycolysis with apoptosis resistance. PFKFB3 enhances glycolytic flux via F2,6BP. PKM2 undergoes alternative splicing and post‐translational modifications to favour its dimeric/nuclear form, transactivating MYC and Cyclin D1. LDHA, induced by HIF1α/c‐Myc, produces lactate, which drives H3K18 lactylation and GPR81 signalling to promote immune evasion. Concurrently, acetylCoA derived from pyruvate fuels lipid synthesis and H3K27 acetylation, up‐regulating PDL1. This enzyme–metabolite network sustains aerobic glycolysis and immune reprogramming in GC.

In GC cells, the Warburg phenotype is driven by a set of core glycolytic enzymes whose expression and functions are uniquely altered. Key glycolytic enzymes—HK2, PFKFB3, PKM2 and LDHA—exhibit aberrant expression in GC that is closely associated with malignant progression and therapeutic resistance.^145^, ^146^, ^147^ Beyond catalytic functions, these enzymes influence tumour cell behaviour through non‐canonical signalling, subcellular localisation dynamics and intercellular communication via exosomes (Figure 2).

#### 3.1.1 HK2

HK2 is the predominant up‐regulated hexokinase isoform in GC, where its overexpression strongly correlates with tumour aggressiveness, poor prognosis and drug resistance.^148^, ^149^ Unlike other HK family members, HK2 possesses a distinctive ability to integrate metabolic flux with apoptosis suppression.

A multi‐layered regulatory network governs its expression in GC. At the transcriptional level, circCUL3/miRNA‐515‐5p disinhibits STAT3 to transactivate HK2.^116^ Post‐transcriptionally, the m6A methyltransferase WTAP stabilises HK2 mRNA by binding its 3′‐UTR m6A site. Post‐translationally, the circNRIP1/miRNA‐149‐5p axis activates AKT1/mTOR signalling to enhance HK2 enzymatic activity.^150^, ^151^ At the protein level, AKT signalling converges on HK2, phosphorylating it at Thr473. This modification is not merely catalytic; it anchors HK2 to the voltage‐dependent anion channels on the outer mitochondrial membrane. This strategic localisation provides HK2 with privileged access to mitochondrial ATP to drive glycolysis while simultaneously inhibiting the release of pro‐apoptotic factors, thereby rendering cells resistant to apoptosis.^149^

Beyond mitochondrial protection, HK2 functions as a metabolic hub. Under conditions of glucose deprivation, it can suppress mTORC1 activity to induce autophagy, promoting cell survival.^149^ Critically, HK2 also engages in a physical interaction with the scaffold protein PDLIM1 in the cytosol. This HK2–PDLIM1 complex potently activates the Wnt/β‐catenin signalling pathway, creating a positive feedback loop that amplifies glycolytic flux and drives GC cells invasion and metastasis.^56^ Thus, HK2 concurrently sustains the energy demands, survival signals and invasive properties of GC cells, making it a highly promising yet complex therapeutic target.

#### 3.1.2 PFKFB3

PFKFB3 is constitutively overexpressed in 81.3% of GC tissues (*p* < .001), with elevated expression strongly associated with lymph node metastasis and poor prognosis (HR = 2.13).^152^, ^153^ Its overexpression is more moderate than in breast or lung cancers, potentially attributable to high baseline PFKFB levels in normal gastric tissue.^152^, ^153^ Its regulation in GC is multi‐layered. Hypoxia drives its transcription via HIF‐1α, while the down‐regulation of the circadian repressor RORα further contributes to its overexpression.^152^, ^154^ Post‐transcriptionally, the miRNA‐3662/CAB39L/AMPK axis provides additional fine‐tuning.^155^

PFKFB3's role in GC extends far beyond simply accelerating glycolysis. It executes a non‐canonical function critical for redox balance and drug resistance: PFKFB3 directly binds to the cystine/glutamate antiporter SLC7A11/xCT and induces its dephosphorylation at Ser26. This interaction suppresses lipid peroxidation‐dependent ferroptosis, establishing a direct link between the glycolytic amplifier and the suppression of an iron‐dependent cell death pathway. This mechanism offers a novel explanation for how GC cells evade the lethal effects of cisplatin‐induced oxidative stress.^156^ Therapeutically, the small‐molecule inhibitor PFK15 potently suppresses this axis, inducing cell cycle arrest and mitochondrial‐dependent apoptosis in GC models, validating PFKFB3 as a metabolic vulnerability.^157^

#### 3.1.3 PKM2

PKM2 is significantly overexpressed in GC and associated with poor OS.^158^, ^159^ Its heterogeneity in GC arises from three interconnected dimensions: expression regulation, structural plasticity and microenvironmental propagation.

Multiple oncogenic axes converge to elevate PKM2 in GC. The PI3K–AKT–mTOR and mTOR–STAT3–c‐Myc cascades up‐regulate PKM2, and pharmacological PI3K inhibition with LY294002 reduces PKM2 levels and lactate output.^85^, ^160^ H. pylori CagA drives ERK‐dependent PKM2 up‐regulation, linking infection‐associated GC to a distinctive glycolytic program.^159^ At the level of alternative splicing, the balance between PTBP1/hnRNPA1 (which favour PKM2) and SRSF3 (which favours PKM1) determines isoform output. In GC, lncRNA CCAT1 stabilises PTBP1 to skew splicing towards PKM2 and amplify glycolytic flux,^90^ whereas LINC00589 promotes hnRNPA1 degradation, curtailing PKM2 production and suppressing peritoneal metastasis.^161^

Once expressed, PKM2's function is dynamically controlled by its conformational state. It oscillates between a catalytically active tetramer and a low‐activity dimer; the dimer favours anabolic metabolism and permits nuclear translocation.^162^, ^163^ KAT2A‐mediated succinylation at K475 disrupts tetramer formation, locking PKM2 in a dimeric state that supports biomass accumulation.^159^ β‐arrestin 1 prevents tetramer assembly while enhancing nuclear PKM2‐driven transcription of MYC and Cyclin D1, coupling metabolic flux to proliferation.^164^ In the nucleus, PKM2 phosphorylates histones (e.g., H3T11) and facilitates HIF‐1α‐dependent transcription, directly linking metabolic rewiring to gene expression.^31^

PKM2 also propagates its influence through exosomal transfer. GC‐derived exosomal PKM2 activates NF‐κB signalling in CAFs, driving IL‐6 and IL‐8 secretion,^165^ and reprograms macrophage lipid metabolism via SCAP–SREBP1 to promote M2 polarisation.^166^, ^167^ Clinically, circulating exosomal PKM2 levels are significantly elevated in GC and demonstrate superior diagnostic performance to CEA and CA19‐9.^167^ Through these multilayered mechanisms, PKM2 establishes itself as a central architect of the metabolic heterogeneity underlying the Warburg effect in GC.

#### 3.1.4 LDHA

LDHA is consistently up‐regulated in GC and recognised as an independent prognostic factor.^168^, ^169^, ^170^ Its expression is governed by an intricate regulatory network that reflects the adaptive plasticity of GC cells.

At the transcriptional level, HIF‐1α functions as the primary activator, while FOXO4 acts as a repressor by directly binding to the LDHA promoter; hypoxia‐induced down‐regulation of FOXO4 is a critical driver of the glycolytic shift in GC.^62^ HOXA9 enhances LDHA transcription via the c‐Myc axis, promoting lymphatic metastasis.^86^ At the post‐transcriptional level, the m6A reader insulin‐like growth factor II mRNA‐binding protein 3 (IGF2BP3) recognises and stabilises LDHA mRNA, fostering lactate accumulation and subsequent immune evasion.^14^ Post‐translationally, LDHA stability is finely tuned by succinylation: lysine‐222 succinylation prevents its lysosomal degradation, a modification markedly elevated in GC tissues and correlated with tumour progression.^171^

In addition to its canonical role in glycolysis, LDHA promotes aggressive GC phenotypes through several non‐metabolic functions. It triggers EMT by up‐regulating ZEB2, thereby enhancing the invasive potential of intestinal‐type GC.^172^ LDHA‐driven lactate accumulation activates the G‐protein‐coupled receptor 81 (GPR81) receptor to modulate CX3CL1, leading to the recruitment of regulatory T cells and the establishment of an immunosuppressive niche.^173^ A further dimension of LDHA function is exemplified by the novel circRNA‐encoded protein SEMA3C‐319aa, which binds to LDHA and modulates glutathione metabolism to induce ferroptosis, implicating LDHA in the regulation of oxidative stress‐induced cell death.^174^

The clinical significance of these findings is well established. Serum LDH levels serve as an independent prognostic factor in patients with unresectable advanced GC, with elevated LDH predicting shorter OS.^170^ A meta‐analysis of 17 studies involving 3842 patients further substantiates that high serum LDH is a robust risk factor for poor OS and progression‐free survival (PFS) in GC patients receiving immunotherapy, with pooled HRs of 2.01 and 2.23, respectively.^16^

### 3.2 Metabolites as signalling molecules

Glycolytic metabolites in GC‐particularly lactate and acetyl‐CoA‐have evolved beyond mere metabolic intermediates into multidimensional signalling hubs that drive malignant progression through microenvironmental remodelling, epigenetic modifications and metabolic symbiosis.

Lactate is a prime example of this functional duality. Through trans‐cellular shuttling via MCT4 and MCT1, lactate establishes a ‘metabolic symbiosis’ within the TME, where glycolytic and oxidative cancer cell populations trade lactate as fuel.^175^, ^176^ Simultaneously, the acidic microenvironment corrupts normal stroma; lactate secreted by GC cells can transform bone marrow‐derived mesenchymal stem cells (BM‐MSCs) into a pro‐tumourigenic inflammatory CAFs‐like phenotype that secretes IL‐8 and enhances PD‐L1 expression.^13^, ^177^ This acidification also directly blunts anti‐tumour immunity by causing metabolic exhaustion in effector CD8‐positive T cells, inhibiting their production of IFN‐γ and IL‐2.^14^, ^178^ Moreover, lactate rewires stress resistance, activating the AMPK–SCD1 signalling cascade and altering lipid metabolism to confer ferroptosis resistance, allowing cancer cells to survive under both metabolic and therapeutic pressures.^179^, ^180^ The end products of this metabolism themselves, serum lactate and pyruvate levels, have been validated as independent predictors of immunotherapy efficacy.^181^

Acetyl‐CoA represents another critical nexus of metabolism and signalling. In the context of the Warburg effect, GC cells reroute pyruvate away from the TCA cycle through PDK2‐mediated inhibition of the PDC. Instead, they generate cytoplasmic acetyl‐CoA via the up‐regulated enzyme ACLY.^182^, ^183^ This metabolic rewiring serves two critical functions. First, the pool of acetyl‐CoA fuels de novo lipid synthesis, providing the building blocks for the membrane biogenesis required for rapid cell division.^184^ Second, and more profoundly, fluctuations in acetyl‐CoA levels directly feed back to the nucleus and epigenome. A reduced glycolytic flux can paradoxically elevate acetyl‐CoA levels, which serves as the acetyl donor for histone modifications; this resulting H3K27 hyperacetylation at the PD‐L1 promoter up‐regulates its expression.^185^ This creates a mechanistic bridge between metabolic flux and the tuning of an immune checkpoint, highlighting how metabolism can dynamically shape tumour immunogenicity.

### 3.3 Warburg effect on the GC microenvironment

#### 3.3.1 Immune cells

##### T cells

Aerobic glycolysis is a vital metabolic pathway that supports T cells growth, proliferation, activation and effector function. T cells preferentially metabolise glucose via aerobic glycolysis to meet the energetic and biosynthetic demands of clonal expansion,^186^ and glucose uptake and utilisation constitute the critical initiating steps for T cells effector responses. T cells activation is tightly regulated by the PI3K/AKT/mTOR signalling pathway. AKT modulates multiple T cells metabolic enzymes, including Glut1, hexokinase 1/2 (HK1/HK2) and pyruvate kinase isozymes M1 and M2, in part through the mTOR complexes. mTORC1 up‐regulates these enzymes, whereas mTORC2 exerts an opposing effect. In particular, mTORC1 up‐regulates Glut1 expression, thereby promoting the initiation of the glycolytic pathway in T cells.^187^, ^188^ GC tissues and cell lines exhibit up‐regulated CD155 expression, which binds to the T cells immunoreceptor with immunoglobulin and ITIM domains (TIGIT) expressed on CD8‐positive T cells. The CD155/TIGIT axis inhibits glucose uptake in CD8‐positive T cells by down‐regulating Glut1 and HK1/HK2, thereby suppressing the AKT/mTOR‐driven glycolytic pathway and reducing IFN‐γ production, ultimately leading to decreased metabolic activity and functional exhaustion.^189^ Hexokinase domain containing 1 (HKDC1), a member of the hexokinase family, plays a key catalytic role in the glycolytic pathway of GC cells. Its expression and activity are regulated by fructose‐1,6‐bisphosphatase (FBPase) and USP15,^190^, ^191^ and it significantly contributes to cellular metabolism and tumour progression.^192^ Elevated HKDC1 expression has been shown in multiple cancer types to impair T cells–mediated immune surveillance and promote immune evasion.^193^, ^194^, ^195^ Although high HKDC1 expression in GC is consistently associated with poor prognosis,^196^ the specific mechanisms by which it shapes the GC immunosuppressive microenvironment and impairs T cells function remain unclear.

**FIGURE 3:**
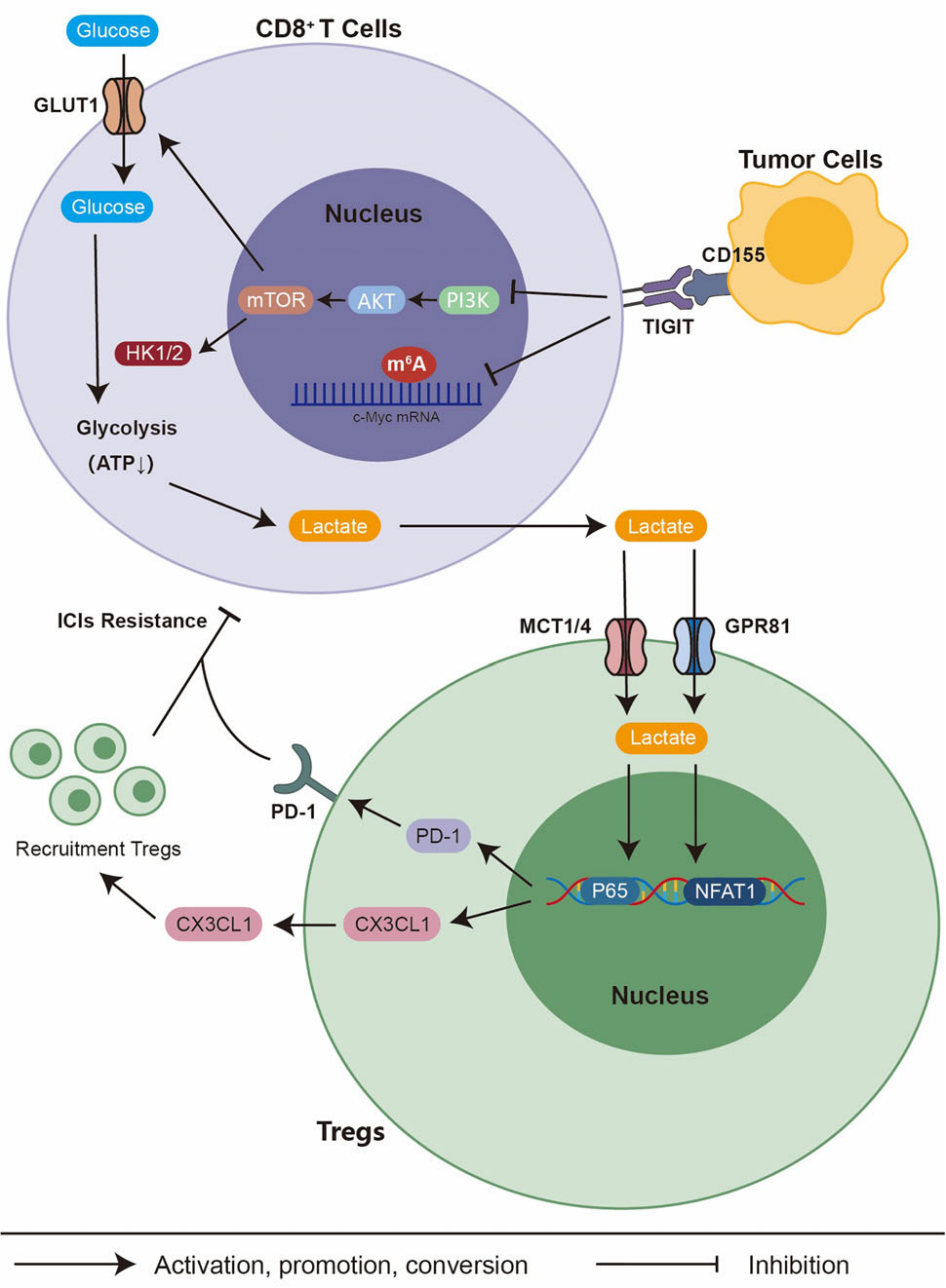
Glycolysis in GC affects T cell immune function. T cells preferentially metabolise glucose to meet their energy needs during clonal expansion. The PI3K/AKT/mTOR signalling pathway can regulate a variety of key enzymes in T cell metabolism, including Glut1 and HK1/HK2, thereby activating T cells. The expression of CD155 in GC cells is up‐regulated, which can induce and bind to the T‐cell immunoreceptor with immunoglobulin and TIGIT on CD8^+^ T cells. CD155/TIGIT signalling can inhibit T cell glycolytic activity and lead to immune failure. Loss of m6A methylase or dysregulation of key m6A enzymes can affect the infiltration of CD8^+^ T cells in the GC microenvironment. Tregs can take up lactate in the microenvironment through MCT1 and GPR81, promoting m6A‐ and NFAT1‐induced enhancement of PD‐1 expression and Tregs recruitment, leading to ICIs resistance.

As a glycolytic tumour, GC exhibits substantial lactic acid (LA) accumulation, which is one of the primary drivers of immunosuppression, invasion and disease progression. This acidic microenvironment is largely regulated by LDHA. GPR81, the physiological receptor for lactate, plays a pivotal role in establishing the lactate‐induced immunosuppressive TME. Lactate/GPR81 signalling induces phosphorylation of NF‐κB p65, thereby promoting the expression of the chemokine CX3CL1 and triggering Tregs recruitment within the TME.^173^ Tregs infiltrating the TME, together with high lactate concentrations, further enhance immunosuppression by inhibiting CD8‐positive T cells cytotoxicity. Furthermore, epigenetic mechanisms can reinforce the Warburg effect, exacerbating lactate accumulation in the GC microenvironment, profoundly disturbing the CD8‐positive T cells/Tregs balance and promoting tumour progression. m6A modification markedly enhances the stability of 3′tRF‐Ala‐AGC, which, by binding to polypyrimidine tract binding protein 1 (PTBP1), boosts glycolytic metabolism and LA production, thereby substantially increasing the proportion of PD‐1‐positive Tregs. In the high‐LA environment generated by the Warburg effect in the GC microenvironment, Tregs actively take up LA via MCT1, promoting NFAT1 nuclear translocation and enhancing PD‐1 expression. Conversely, PD‐1 expression is reduced in CD8‐positive T cells, ultimately contributing to ICIs resistance.^197^ Deficiency of m6A methyltransferases or dysregulation of key m6A‐modifying enzymes can markedly alter CD8‐positive T cells infiltration in the GC microenvironment. Overexpression of IGF2BP3 enhances mRNA stability by binding to m6A sites on LDHA transcripts, leading to LA accumulation in the TME. This, in turn, impairs CD8‐positive T cells cytotoxicity as well as IFN‐γ, TNF‐α and granzyme B production, thereby compromising anti‐tumour immune activity and facilitating immune escape.^14^ The potential role of IGF2BP3 in mediating tumour progression through immunomodulation has recently garnered considerable attention, although it remains insufficiently characterised in GC. For instance, in human cervical carcinoma, IGF2BP3 stabilises the mRNAs of GLS and GLUD1 via m6A‐dependent binding, thereby promoting Tregs‐mediated immune escape.^198^ In hepatocellular carcinoma, IGF2BP3 expression is elevated and positively correlated with macrophage infiltration, and IGF2BP3 binds to TGF‐β1 or CCL5 mRNA to increase their expression, thereby inducing M2 TAMs polarisation and infiltration while suppressing CD8‐positive T cells activity (Figure 3).^199^

In summary, T cells growth, proliferation and effector functions critically depend on glucose uptake and utilisation. Regulation of glycolytic pathways within the heterogeneous GC microenvironment significantly influences T cells‐mediated anti‐tumour immunity. Furthermore, the accumulation of metabolic by‐products such as lactate, as a consequence of the Warburg effect, is a key driver of the immunosuppressive GC microenvironment. Therefore, effective strategies targeting glycolytic pathways in GC are urgently needed to restore anti‐tumour immunity.

##### TAMs

**FIGURE 4:**
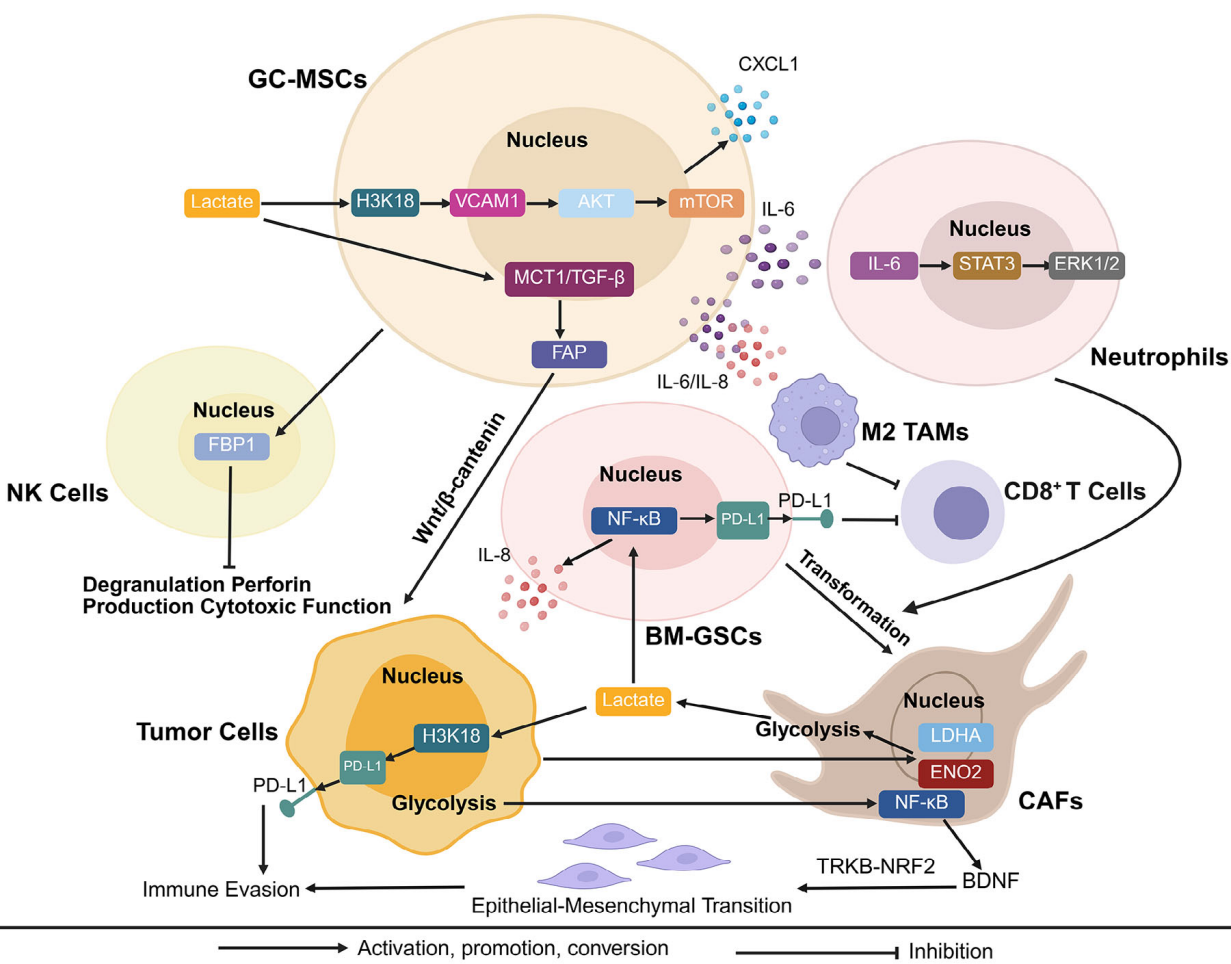
Glycolysis in GC affects microenvironment stromal cells. Lactate accumulation in the tumour microenvironment due to the Warburg effect recruits gastric cancer‐associated mesenchymal stem cells (GC‐MSCs). Lactate induces H3K18 lactylation, up‐regulates VCAM1 transcription, activates the AKT/mTOR signalling pathway and enhances CXCL1 secretion, thereby promoting GC‐MSCs migration to tumour tissues. GC‐MSCs further induce M2 macrophage polarisation and infiltration while inhibiting immune cell responses. Via MCT1/TGF‐β1 signalling, lactate significantly activates GC‐MSCs and up‐regulates FAP expression, which promotes epithelial–mesenchymal transition via the Wnt/β‐catenin pathway to drive gastric cancer progression. Additionally, GC‐MSCs inhibit NK cell glycolytic metabolism by up‐regulating FBP1 expression, impairing their degranulation, perforin production and cytotoxicity to exert immunosuppression. Lactate in the GC microenvironment activates the NF‐κB pathway in bone marrow‐derived mesenchymal stem cells (BM‐MSCs), increasing IL‐8 secretion to promote gastric cancer cell migration/invasion and up‐regulating PD‐L1 to enhance cytotoxicity against CD8‐positive T cells. GC‐MSCs regulate neutrophils via the IL‐6–STAT3–ERK1/2 cascade, and neutrophils induce MSC conversion to cancer‐associated fibroblasts (CAFs), synergistically promoting tumour progression. GC cells induce glycolytic gene (LDHA, ENO2) expression in fibroblasts, shifting their metabolism to aerobic glycolysis. CAFs‐derived lactate induces H3K18 lactylation and PD‐L1 up‐regulation in GC cells, facilitating immune evasion. GC cell‐secreted lactate also guides CAFs to produce BDNF via NF‐κB, activating TrkB‐Nrf2 signalling to promote GC therapeutic resistance.

Glucose addiction in GC cells, together with the acidic microenvironment induced by the Warburg effect, promotes M2 polarisation of macrophages, thereby fostering an immunosuppressive microenvironment and driving tumour progression. Using microencapsulated in vitro cell models that closely recapitulate the TME, TAMs have been shown to rely on glycolysis as a major energy source, thereby inducing the expression of PCNA, VEGF and MMPs in GC cells,^200^ all of which are closely associated with GC invasion and metastasis.^201^, ^202^, ^203^ SLC2A3, which functions as an oncogenic driver, is overexpressed in GC, leading to pronounced glucose addiction and establishing an SLC2A3–STAT3–SLC2A3‐positive feedback loop. This loop activates the STAT3 signalling pathway, up‐regulates downstream glycolytic genes such as SLC2A1, HK2, LDHA and PKM2, promotes macrophage infiltration into the TME and induces their transition to the M2 subtype.^204^ The high‐lactate GC microenvironment further promotes M2 TAMs polarisation via MCT/HIF‐1α signalling, inducing arginase‐1 expression and thereby facilitating tumour angiogenesis and immune escape in GC.^205^, ^206^ A retrospective analysis has identified elevated IL‐4 as a key factor contributing to resistance to anti‐PD‐1 antibody therapy. The IL‐4/IL‐4R axis up‐regulates the PI3K/AKT–mTOR signalling pathway, thereby enhancing aerobic glycolysis and promoting M2 polarisation in macrophages. The resulting acidic microenvironment up‐regulates Fcγ receptor IIB (FcγRIIB) expression on M2 TAMs, which suppresses T cells cytotoxicity and ultimately promotes immune escape (Figure 4).^207^

#### 3.3.2 Stromal cells

##### GC‐MSCs

GC‐MSCs can be isolated from GC tissues. Owing to their multipotent differentiation capacity, GC‐MSCs exert broad immunosuppressive effects within the TME, modulating the activity of cells in both the innate and adaptive immune systems to promote tumour initiation and progression.^208^, ^209^ LA accumulation in the TME, driven by the Warburg effect, plays a crucial role in recruiting GC‐MSCs and thereby promoting GC progression. LA induces H3K18 lactylation, which up‐regulates VCAM1 transcription, activates AKT/mTOR signalling and enhances CXCL1 secretion. These changes collectively promote GC‐MSCs migration into tumour tissues. Concurrently, GC‐MSCs induce M2 TAMs polarisation and infiltration, suppressing anti‐tumour immune responses and thereby accelerating tumour progression.^122^ LA also markedly activates GC‐MSCs and increases fibroblast activation protein (FAP) expression via MCT1/TGF‐β1 signalling.^177^ In addition, lactate promotes EMT through activation of the Wnt/β‐catenin pathway, further driving GC progression.^210^ GC‐MSCs can also induce NK cells to up‐regulate FBP1 expression, thereby inhibiting NK cells glycolytic metabolism and leading to impaired degranulation, reduced perforin production and diminished cytotoxicity, ultimately exerting an immunosuppressive effect (Figure 4).^211^

##### CAFs

Tumour‐derived lactate contributes to CAFs formation and activation by down‐regulating p62 transcription^212^ and up‐regulating its corresponding binding proteins.^213^, ^214^ BM‐MSCs are well established as a major cellular source of CAFs. In the GC microenvironment, lactate can robustly activate the NF‐κB signalling pathway in BM‐MSCs, leading to markedly increased IL‐8 secretion. This, in turn, promotes GC cells migration and invasion and enhances their inhibitory effects on CD8‐positive T cells via PD‐L1 up‐regulation, thereby reinforcing their phenotypic similarity to inflammatory CAFs.^13^ Furthermore, GC‐MSCs modulate and activate neutrophil chemotaxis, survival and effector functions via the IL‐6–STAT3–ERK1/2 signalling cascade, while neutrophils induce MSCs to differentiate into CAFs. These two processes engage in reciprocal crosstalk that synergistically promotes GC progression.^215^ In GC, CAFs play a pivotal role in shaping an immunosuppressive TME by remodelling the extracellular matrix and modulating cytotoxic lymphocyte infiltration into tumour tissues.^216^ Interactions between CAFs and GC cells are profoundly influenced by the glycolytic metabolite lactate. Diffuse‐type GC (DGC) cells with high metastatic potential strongly induce the expression of glycolysis‐related genes, including LDHA and ENO2, in surrounding fibroblasts, thereby promoting a metabolic shift from oxidative phosphorylation to aerobic glycolysis and enhancing glucose consumption as well as lactate production.^217^ CAFs‐derived lactate promotes H3K18 lactylation in GC cells, facilitating direct binding of the assembly factor for spindle microtubules (ASPM) to NCAPG (non‐SMC condensin I complex subunit G) and driving BUB3‐mediated NCAPG deubiquitination. This axis activates the SRC/STAT3 pathway and increases PD‐L1 expression, thereby promoting immune evasion in GC.^15^ LA secreted by GC cells also serves as a key ligand for tropomyosin receptor kinase B (TrkB), directing CAFs to produce brain‐derived neurotrophic factor (BDNF) via an NF‐κB–dependent pathway. The resulting increase in BDNF activates TrkB–Nrf2 signalling, thereby mediating epithelial–mesenchymal interactions in GC cells and promoting therapeutic resistance (Figure 4).^218^

## 4 TARGETING THE WARBURG EFFECT: CLINICAL TRANSLATION

Building on the molecular heterogeneity of the Warburg effect described in preceding sections, translating these insights into clinical practice now demands an integrated strategy that extends beyond single‐target inhibition. The evolving paradigm encompasses three interdependent pillars: a multi‐dimensional biomarker system that captures metabolic status at imaging, circulating and tissue levels; enzyme‐specific therapeutic interventions that exploit isoform selectivity and post‐translational regulation; and rational combination regimens that disrupt metabolic–immune crosstalk to overcome intrinsic and acquired resistance.

### 4.1 Multi‐dimensional biomarkers and prognosis assessment

#### 4.1.1 Imaging biomarkers

^1^ ^8^F‐FDG PET/CT remains the cornerstone for non‐invasive assessment of tumour glycolytic activity. Standardised uptake parameters, including SUVmax, metabolic tumour volume (MTV) and total lesion glycolysis (TLG), consistently correlate with GC invasiveness, pathological stage and OS.^73^, ^170^, ^219^ Here, the oncogenic driver MACC1 exhibits a significant positive correlation with SUVmax, establishing a direct link between a molecular driver of the Warburg effect and its imaging phenotype.^73^ Beyond reflecting overall metabolic burden, functional imaging captures intratumoural heterogeneity, which can inform biopsy targeting.

#### 4.1.2 Circulating metabolic biomarkers

Serum LDH is a robust, broadly accessible prognostic marker. A meta‐analysis of 17 studies involving 3842 patients confirmed that elevated serum LDH independently predicts inferior OS and PFS in GC patients receiving ICIs, with pooled hazard ratios of 2.01 and 2.23, respectively.^16^ Complementing this, direct measurement of serum lactate and pyruvate has emerged as a predictor of anti‐PD‐1/PD‐L1 efficacy: a decline in these metabolites correlates with higher disease control rates and prolonged survival, with predictive accuracy surpassing conventional PD‐L1 immunohistochemical scoring.^181^ Liquid biopsy further expands the biomarker repertoire—plasma exosomal PKM2 is significantly elevated in GC, demonstrates superior diagnostic performance to CEA and CA19‐9, and its levels reflect tumour stage and prognosis.

#### 4.1.3 Gene‐expression‐based risk models

Multi‐gene signatures derived from lactate metabolism‐related genes, such as those centred on the lactate transporter SLC5A12, can stratify patients into distinct prognostic subgroups. These signatures not only predict OS but also forecast responses to adjuvant chemotherapy and immunotherapy, providing a transcriptomic complement to circulating metabolite measurements.^167^, ^176^, ^220^

#### 4.1.4 Tissue metabolic biomarkers

At the tissue level, biomarkers are extending beyond enzyme expression to post‐translational modifications. H3K18la, a direct epigenetic consequence of lactate accumulation, is enriched in GC and associated with adverse outcomes.^103^ This mark is dynamically regulated by the delactylase SIRT1; loss of SIRT1 activity amplifies a H19–LDHA–lactylation‐positive feedback loop, thereby reinforcing glycolytic flux and tumour progression.^125^ Furthermore, the HSP90‐chaperoned glycolytic enzyme complex (comprising PGK1, PKM2, ENO1 and LDHA) yields a quantitative HGScore that independently correlates with chemoresistance and poor prognosis.^221^ Even the gut microbiota contributes metabolic information: GC patients show decreased plasma propionate and butyrate alongside increased TCA cycle intermediates, offering a non‐invasive approach for early detection.^222^ To make the translational maturity more transparent, we classified representative Warburg effect‐related biomarkers and therapeutic strategies using an evidence hierarchy, ranging from direct GC clinical evidence to conceptual or early mechanistic evidence. The resulting evidence‐level summary is presented in Table 2. Specifically, metabolic imaging and serum LDH currently have relatively established prognostic feasibility, serum lactate/pyruvate and exosomal PKM2 are promising but require external validation, whereas H3K18la, gene‐expression signatures and most therapeutic strategies remain exploratory or preclinical.

**TABLE 2:** Translational evidence levels of representative Warburg effect‐related biomarkers and therapeutic strategies in gastric cancer.

Category	Biomarker/therapeutic strategy	Main evidence source	GC‐specificity	Translational evidence level	Translational maturity and major limitations	References
Imaging biomarker	18F‐FDG PET/CT parameters, including SUVmax, MTV and TLG	Clinical imaging studies and molecular correlation studies	Direct GC clinical evidence	Clinical cohort evidence	Relatively close to clinical use for metabolic burden assessment and risk stratification. Limitations include retrospective designs, nonuniform cutoff values, heterogeneous imaging protocols and limited prospective validation for immunotherapy prediction.	^16^, ^73^
Circulating biomarker	Serum LDH and LDH‐based indices, including LDH‐to‐albumin ratio	GC clinical cohorts and meta‐analysis in patients receiving ICIs	Direct GC clinical evidence	Meta‐analysis and clinical cohort evidence	Relatively mature and clinically accessible prognostic biomarker. However, LDH is nonspecific and can be affected by tumour burden, inflammation, liver injury, haemolysis and performance status.	^16^, ^17^, ^169^, ^170^
Circulating metabolic biomarker	Serum lactate and pyruvate	Prospective biomarker study in advanced GC treated with anti‐PD‐1/PD‐L1 therapy	Direct GC prospective clinical evidence	Prospective GC biomarker evidence	Promising predictor of immunotherapy response. Larger multi‐centre validation is required, and assay timing, dynamic thresholds and integration with PD‐L1, MSI and EBV status remain unclear.	^181^
Liquid biopsy biomarker	Plasma exosomal PKM2	GC clinical biomarker study and mechanistic studies linking exosomal PKM2 to macrophage and CAF metabolic reprogramming	Direct GC clinical and preclinical evidence	Clinical association plus mechanistic evidence	Promising non‐invasive diagnostic and prognostic biomarker. Exosome isolation, normalisation and quantification methods require standardisation; independent external validation remains limited.	^165^, ^166^, ^167^
Gene‐expression model	Lactate metabolism‐related gene signatures, including SLC5A12‐centred models and lactate metabolism subtypes	Transcriptomic modelling using GC datasets with retrospective validation	Direct GC retrospective bioinformatic evidence	Retrospective clinical‐transcriptomic evidence	Useful for exploratory patient stratification and therapy–response prediction. Prospective validation, platform harmonisation and conversion into deployable clinical assays are required.	^176^, ^220^
Tissue epigenetic biomarker	H3K18 lactylation and lactylation‐related markers	GC tissue studies, lactylome profiling and functional mechanistic studies	Direct GC tissue and preclinical evidence	Tissue cohort plus mechanistic evidence	Biologically compelling marker linking lactate accumulation to epigenetic remodelling and immune escape. No standardised IHC scoring system is available, and its predictive value for treatment response remains uncertain.	^18^, ^19^, ^103^, ^112^, ^119^, ^120^, ^121^, ^122^, ^123^, ^124^, ^125^
Tissue metabolic score	HSP90‐chaperoned glycolytic output complex/HGScore	GC tissue cohort and functional studies	Direct GC tissue and preclinical evidence	Clinical association plus mechanistic evidence	Potential marker of chemoresistance and poor prognosis. Multi‐centre validation and standardised quantification are needed before clinical implementation.	^221^
Plasma metabolomic biomarker	Short‐chain fatty acids and TCA‐cycle intermediate profile	Human plasma metabolomic study in GC	Direct GC clinical metabolomic evidence	Clinical metabolomic association	Potential non‐invasive approach for early detection or metabolic stratification. Diagnostic thresholds, reproducibility and specificity versus benign gastric disease require further validation.	^222^
Therapeutic strategy	HK2‐directed glycolytic inhibition, including 2‐DG, 3‐BrPA and HK2‐regulatory targeting	GC preclinical studies and mixed‐tumour early‐phase clinical trial of 2‐DG	Direct GC preclinical plus indirect or mixed clinical evidence	Preclinical GC evidence plus early clinical mixed‐tumour evidence	Mechanistically strong but not ready for routine GC treatment. Major barriers include dose‐limiting systemic toxicity, limited HK2 selectivity, QTc prolongation with 2‐DG and metabolic compensation.	^97^, ^219^, ^223^, ^224^, ^226^, ^233^
	PFKFB3 inhibition, including PFK15/PFK‐158 and combination with ferroptosis induction or targeted therapy	GC preclinical studies and mixed solid‐tumour early‐phase trial	Direct GC preclinical plus indirect clinical evidence	Preclinical GC evidence plus indirect early clinical evidence	Promising in chemoresistance and metabolic vulnerability contexts. GC‐specific clinical trials are lacking, and optimal combination partners, dose, schedule and predictive biomarkers remain undefined.	^156^, ^157^, ^255^
	PKM2 modulation, including inhibition, allosteric activation and alternative‐splicing regulation	Preclinical studies in GC and other cancers	Mainly preclinical evidence	Preclinical and conceptual evidence	Mechanistically attractive because PKM2 acts as both metabolic enzyme and transcriptional regulator. Dual functional states of PKM2 complicate therapeutic direction; clinical translation remains limited.	^161^, ^162^, ^163^, ^164^, ^228^, ^229^
	LDHA inhibition and lactate‐output suppression, including oxamate, catechin and related approaches	GC cell and preclinical chemoresistance models	Direct GC preclinical evidence	Cell‐line and preclinical evidence	Potential strategy to reverse lactate‐driven chemoresistance and immune suppression. Systemic LDHA inhibition may affect normal tissues and immune cells, and mature GC clinical evidence is lacking.	^168^, ^171^, ^172^, ^173^, ^174^, ^231^, ^232^
	MCT1/MCT4‐mediated lactate transport inhibition, including AZD3965 and MCT4 blockade	First‐in‐human AZD3965 trial in advanced cancers and preclinical immunotherapy‐combination models	Indirect mixed solid‐tumour clinical evidence and preclinical evidence	Indirect early clinical evidence	Biomarker‐selected strategy may be feasible, particularly in MCT1‐high/MCT4‐low tumours. Limitations include limited monotherapy efficacy, MCT4‐mediated compensation and absence of GC‐specific cohorts.	^44^, ^45^
	Metabolic modulation combined with immunotherapy, including DCA‐ or lactate‐axis‐directed approaches	Mechanistic studies and non‐GC or mixed clinical evidence	Mostly indirect or preclinical evidence	Preclinical GC rationale plus indirect clinical evidence	Conceptually promising for reversing acidic, lactate‐rich, immunosuppressive TME. Systemic glycolytic modulation may impair effector T cells function; pharmacodynamic biomarker monitoring is required.	^173^, ^178^, ^185^, ^197^, ^234^, ^235^ ^,^ ^245^, ^247^, ^283^, ^284^, ^285^
	Natural compounds with anti‐glycolytic activity, including curcumin, wogonin, baicalein, licochalcone A, helichrysetin, oleanolic acid and tanshinone IIA	GC preclinical studies	Direct GC preclinical evidence	Preclinical and lead‐compound evidence	Provides lead structures for metabolic intervention. Low bioavailability, uncertain target specificity, formulation challenges and lack of clinical‐grade validation limit translation.	^232^
	Mitochondrial metabolic inhibition, including metformin, ME‐344 and devimistat‐related approaches	Non‐GC clinical trials and mixed solid‐tumour studies	Indirect non‐GC or mixed‐tumour evidence	Indirect clinical evidence	Useful for hypothesis generation and combination design. No GC‐specific efficacy signal is available, and evidence is heterogeneous across cancer types, doses and treatment contexts.	^236^, ^237^, ^238^, ^239^, ^240^, ^241^, ^242^, ^243^, ^248^, ^249^, ^250^, ^251^

### 4.2 Therapeutic strategies targeting glycolytic enzymes

**FIGURE 5:**
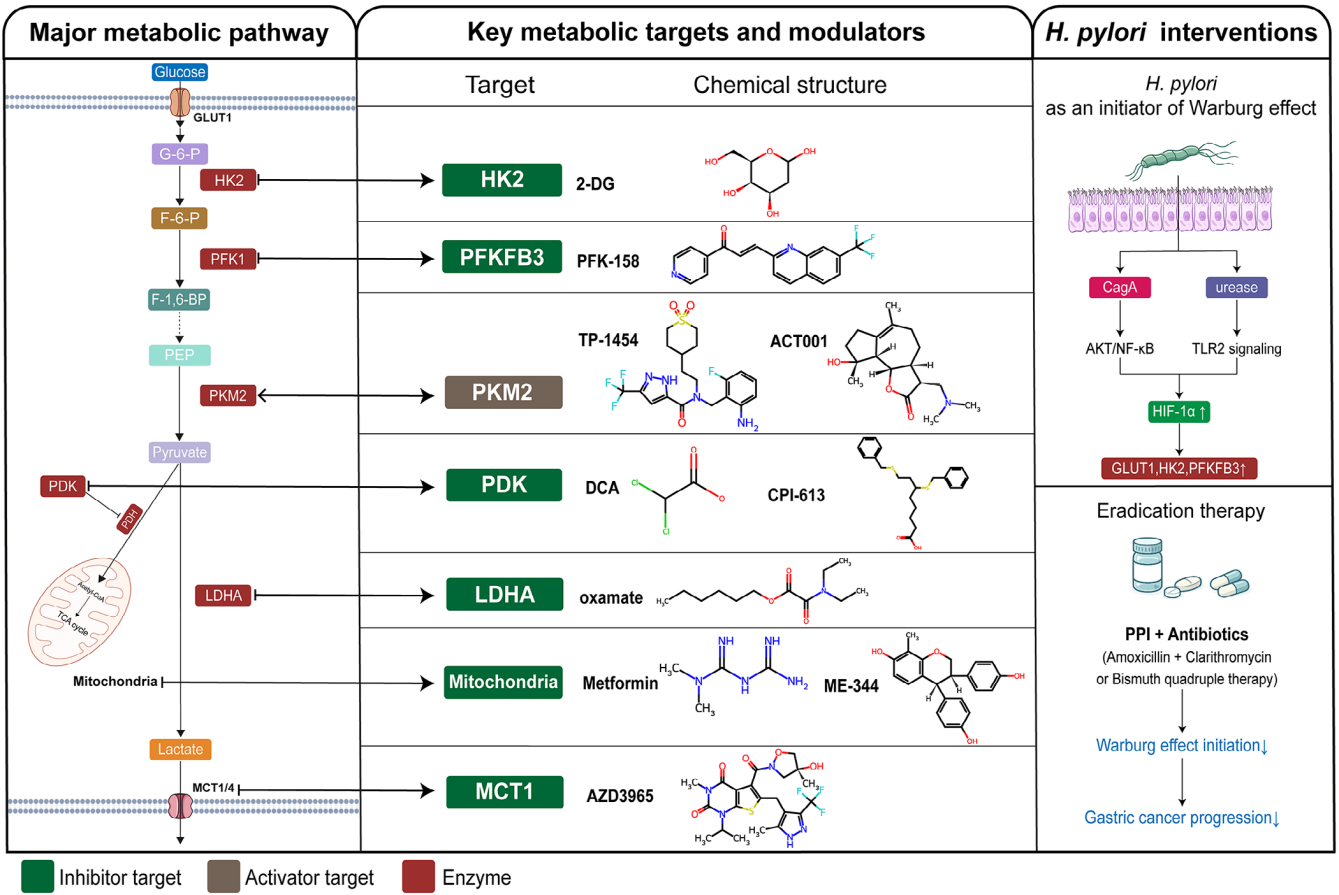
Schematic overview of major Warburg effect‐related metabolic pathways, therapeutic targets, representative metabolic modulators and *H. pylori*‐directed interventions in gastric cancer. Glucose uptake through GLUT1 fuels glycolytic flux through HK2, PFK1/PFKFB3 and PKM2, followed by pyruvate conversion, PDK/PDH‐regulated mitochondrial entry, LDHA‐mediated lactate production and MCT1/4‐dependent lactate export. Representative druggable nodes and corresponding compounds include HK2/2‐DG, PFKFB3/PFK‐158, PKM2 modulators TP‐1454 and ACT001, PDK‐ or mitochondrial metabolism‐targeting agents DCA, CPI‐613, metformin and ME‐344, LDHA/oxamate, and MCT1/AZD3965. *H. pylori* virulence factors, including CagA and urease, can activate AKT/NF‐κB or TLR2 signalling, converge on HIF‐1α and enhance glycolysis‐related effectors such as GLUT1, HK2 and PFKFB3, whereas eradication therapy may attenuate infection‐driven Warburg effect initiation and gastric cancer progression.

A schematic overview of the major Warburg effect‐related metabolic pathways, proposed therapeutic targets, representative metabolic inhibitors and *H. pylori*‐directed interventions is provided in Figure 5.

#### 4.2.1 HK2 inhibitors

HK2 inhibition has been explored using 2‐DG, a competitive glucose analogue, and 3‐bromopyruvate (3‐BrPA), an alkylating agent that also triggers mitochondrial apoptosis.^219^, ^223^, ^224^, ^225^ The combination of 3‐BrPA with sodium citrate produces synergistic anti‐proliferative effects through survivin down‐regulation and Bax/caspase pathway activation.^224^ However, clinical advancement of these agents has been hampered by a lack of HK2 selectivity and dose‐limiting systemic toxicities. A more targeted strategy emerges from understanding HK2 post‐translational regulation: the PIM2 kinase phosphorylates HK2 at Thr473, enhancing both its protein stability and enzymatic activity, thereby promoting glycolysis and paclitaxel resistance. Pharmacological inhibition of PIM2 with SMI‐4a reverses this phosphorylation‐driven resistance.^226^ Critically, the therapeutic feasibility of HK2 targeting is strongly supported by genetic evidence: systemic, whole‐body deletion of HK2 in adult mice effectively suppresses tumour initiation and maintenance without inducing significant physiological dysfunction, establishing HK2 as a tumour‐selective vulnerability.^219^

#### 4.2.2 PFKFB3 inhibitors

The small‐molecule inhibitor PFK15 arrests GC cells proliferation by disrupting cyclin‐dependent kinase signalling and inducing mitochondrial‐dependent apoptosis, while exhibiting favourable pharmacokinetic properties including reduced clearance compared with its predecessor 3PO. Beyond its canonical metabolic role, PFKFB3 suppresses ferroptosis through interaction with the cystine/glutamate transporter SLC7A11/xCT; consequently, combining PFKFB3 inhibition with the ferroptosis inducer erastin synergistically potentiates cisplatin cytotoxicity.^156^ At the regulatory RNA level, miRNA‐613 directly targets PFKFB2, another family member, thereby suppressing the Warburg effect and inhibiting GC cells proliferation and invasion.^227^

#### 4.2.3 PKM2 modulators

The therapeutic targeting of PKM2 is complicated by its dual functional states: a low‐activity dimeric form that facilitates anabolic metabolism and a high‐activity tetrameric form that drives ATP production.^163^ Both states can be therapeutically exploited. Shikonin, a PKM2 inhibitor, suppresses glycolytic flux and induces apoptosis.^228^ In contrast, the allosteric activator DASA‐58 forces tetramerisation, yet also impairs tumour growth,^229^ likely by diverting glucose carbon away from biosynthesis. This duality positions PKM2 as a metabolic rheostat that can be modulated in either direction depending on the metabolic context. Post‐transcriptional control of PKM2 alternative splicing offers another intervention node: the lncLINC00589 promotes ubiquitin‐mediated degradation of hnRNPA1, a splicing factor required for PKM2 isoform generation; loss of hnRNPA1 shifts splicing towards the PKM1 isoform, reduces glycolytic capacity and inhibits peritoneal metastasis.^161^

#### 4.2.4 LDHA inhibitors

 LDHA catalyses the terminal step of glycolysis, and its inhibition not only reduces lactate production but also disrupts redox homeostasis. The competitive inhibitor oxamate decreases lactate accumulation, induces mitochondrial ROS and triggers apoptosis.^230^, ^231^ ILDHA inhibition has been explored as a strategy to disrupt glycolytic dependence in GC. Suppression of LDHA reduces malignant phenotypes in human GC cells, supporting LDHA as a potential metabolic vulnerability.^231^ It is noteworthy that LDHA also possesses non‐metabolic functions: it promotes EMT through transcriptional up‐regulation of ZEB2, thereby enhancing the invasive capacity of intestinal‐type GC.^172^

#### 4.2.5 Natural compounds

A diverse array of plant‐derived natural products has demonstrated anti‐glycolytic activity in GC models. These include curcumin, which activates p53 through ROS induction; wogonin and baicalein, which inhibit HIF‐1α; licochalcone A, which suppresses the Akt/HK2 axis; helichrysetin, which targets c‐Myc/PDHK1; oleanolic acid, which reduces HK2 and PFK1 expression via YAP/HIF‐1α; and tanshinone IIA, which down‐regulates G6PI and LDHB.^232^ While these compounds provide valuable lead structures, their clinical translation requires overcoming limitations in bioavailability and target selectivity, potentially through nanoformulation strategies. Representative preclinical agents and intervention strategies targeting glycolytic vulnerabilities in GC are summarised in Table 3, with their putative mechanisms and reported in vitro or in vivo activity.

**TABLE 3:** Representative preclinical glycolysis‐targeting agents in gastric cancer models.

Target/pathway	Agent/strategy	Proposed mechanism	Preclinical model/evidence source	Reported in vitro activity	Reported in vivo activity	Translational notes/limitations	References
HK2/glucose utilisation	2‐deoxy‐D‐glucose (2‐DG)	Competitive glucose analogue that blocks glycolytic flux through interference with glucose utilisation and HK‐dependent phosphorylation.	GC‐related preclinical rationale and early mixed‐tumour clinical experience; also used as a pharmacologic glycolysis inhibitor in GC‐MSC‐driven chemoresistance models.	Restrains glycolysis‐dependent survival and has been reported to attenuate chemotherapy resistance in glycolysis‐addicted GC contexts.	Specific GC in vivo efficacy is not consistently established in the summarised evidence; clinical use is limited by systemic exposure.	Useful proof‐of‐concept glycolytic inhibitor, but limited by lack of tumour selectivity, systemic toxicity and QTc prolongation in early clinical testing.	^97^, ^219^, ^223^, ^233^
HK2/glycolysis and mitochondrial apoptosis	3‐Bromopyruvate (3‐BrPA)	Alkylating glycolytic inhibitor that targets HK2‐associated glycolysis and can trigger mitochondrial apoptosis.	GC cell‐based preclinical studies; combination with sodium citrate examined as a sensitising approach.	3‐BrPA combined with sodium citrate produces synergistic anti‐proliferative effects, associated with survivin down‐regulation and Bax/caspase pathway activation.	Robust GC‐specific in vivo activity is not emphasised in the reviewed section.	Mechanistically attractive but non‐specific; clinical translation is constrained by toxicity, delivery and selectivity concerns.	^224^
PFKFB3/fructose‐2,6‐bisphosphate production	PFK15	Small‐molecule PFKFB3 inhibitor that suppresses the glycolytic amplifier controlling fructose‐2,6‐bisphosphate production.	GC cell models and PFKFB3‐dependent metabolic vulnerability studies.	Arrests GC cell proliferation by disrupting cyclin‐dependent kinase signalling and inducing mitochondrial‐dependent apoptosis.	Favourable pharmacokinetic properties relative to 3PO are noted, but GC‐specific in vivo efficacy requires further validation.	Promising preclinical inhibitor; optimal combination partners, dose schedule and predictive biomarkers remain undefined.	^157^
PFKFB3‐SLC7A11/xCT/ferroptosis defence	PFKFB3 inhibition + erastin	PFKFB3 interacts with SLC7A11/xCT and suppresses ferroptosis; combining PFKFB3 inhibition with erastin relieves this defence mechanism.	GC cisplatin‐resistance and ferroptosis‐related preclinical models.	Synergistically potentiates cisplatin cytotoxicity and promotes ferroptosis‐related vulnerability.	In vivo confirmation and therapeutic window remain insufficiently established.	Supports combination rather than monotherapy; ferroptosis status and cystine/glutamate transport markers may guide selection.	^156^
PKM2 conformational regulation	DASA‐58	Allosteric PKM2 activator that forces tetramerisation, thereby shifting glucose carbon away from biosynthesis.	Preclinical PKM2‐modulation models.	Impairs tumour growth‐related metabolic programs by stabilising the high‐activity tetrameric state.	In vivo activity is described as impaired tumour growth in preclinical models, but GC‐specific validation remains limited.	Highlights that PKM2 can be modulated by activation rather than inhibition; patient selection should consider PKM2 state/function.	^229^
PKM alternative splicing	LINC00589‐based splicing intervention	Promotes ubiquitin‐mediated degradation of hnRNPA1, shifting PKM splicing from PKM2 towards PKM1 and reducing glycolytic capacity.	GC peritoneal metastasis‐related preclinical models.	Reduces glycolytic capacity by limiting PKM2 isoform generation.	Inhibits peritoneal metastasis in preclinical GC models.	Not a conventional small‐molecule drug; delivery and RNA‐targeting feasibility require substantial development.	^161^
LDHA/lactate production and redox balance	Oxamate	Competitive LDHA inhibitor that decreases lactate production and perturbs NAD+/NADH‐linked redox homeostasis.	GC cell models assessing LDHA suppression.	Suppresses malignant phenotypes in human GC cells through LDHA‐related metabolic vulnerability.	GC‐specific in vivo activity is not emphasised in the summarised evidence.	Useful LDHA proof‐of‐concept agent, but potency, specificity and systemic metabolic effects limit direct translation.	^168^, ^231^
LDHA/chemotherapy resistance	Catechin	Natural flavonoid reported to target LDHA and suppress lactate‐driven resistance programs.	Natural‐compound evidence summarised in GC glycolysis‐focused review.	Reported as a natural‐product lead linked to LDHA/glycolysis modulation in GC‐related preclinical literature.	Direct GC in vivo confirmation and clinically achievable exposure remain uncertain.	Represents a natural‐product lead rather than a clinically validated LDHA inhibitor; bioavailability and specificity are major limitations.	^232^
HIF‐1alpha/glycolysis	Wogonin and baicalein	Natural flavonoids that inhibit HIF‐1alpha‐associated glycolytic regulation.	GC preclinical models summarised under natural compounds.	Reduce glycolysis‐associated malignant phenotypes through HIF‐1alpha suppression.	GC in vivo efficacy and pharmacokinetic robustness require further validation.	Lead compounds with limited clinical readiness; target specificity and formulation need optimisation.	^232^
Akt‐HK2 axis	Licochalcone A	Suppresses Akt/HK2 signalling to reduce glycolytic activity.	GC preclinical models summarised under natural compounds.	Inhibits glycolysis‐associated tumour cell growth and survival programs.	In vivo efficacy is not consistently established in the summarised text.	Potential lead compound; translation limited by bioavailability, potency and target specificity.	^232^
c‐Myc/PDHK1 metabolic axis	Helichrysetin	Targets c‐Myc/PDHK1 signalling, thereby reducing Warburg‐type glycolytic programming.	GC preclinical models summarised under natural compounds.	Suppresses glycolysis‐associated malignant activity in GC model systems.	Further in vivo and pharmacologic validation are needed.	Exploratory natural compound; requires optimisation before clinical translation.	^232^
YAP/HIF‐1alpha‐HK2/PFK1 axis	Oleanolic acid	Reduces HK2 and PFK1 expression through YAP/HIF‐1alpha‐related regulation.	GC preclinical models summarised under natural compounds.	Dampens glycolytic enzyme expression and glycolysis‐linked tumour phenotypes.	GC‐specific in vivo evidence and clinically achievable dosing need further clarification.	Potential lead structure; formulation and patient selection remain challenges.	^232^
G6PI/LDHB‐related glycolysis	Tanshinone IIA	Down‐regulates G6PI and LDHB to suppress glycolytic metabolism.	GC preclinical models summarised under natural compounds.	Reduces glycolysis‐associated malignant phenotypes.	In vivo evidence and pharmacodynamic biomarkers remain insufficient.	Exploratory natural product; requires target validation, improved delivery and toxicity assessment.	^232^
p53/ROS‐glycolysis crosstalk	Curcumin	Activates p53 through ROS induction and suppresses glycolysis‐associated tumour programs.	GC preclinical models summarised under natural compounds.	Inhibits glycolysis‐related proliferation/survival programs.	In vivo translation is limited by low bioavailability despite broad preclinical activity.	Requires formulation strategies such as nanoformulation to improve exposure and specificity.	^232^

#### 4.2.6 Clinical trial landscape

The clinical development of glycolytic inhibitors has encountered consistent challenges. Phase I trials have revealed narrow therapeutic indices, as exemplified by 2‐DG, which was limited by QTc interval prolongation.^233^ Monotherapy with agents such as the PFKFB3 inhibitor PFK‐158 or the MCT1 inhibitor AZD3965 has yielded modest anti‐tumour activity in unselected patient populations.^44^ Notably, the AZD3965 trial suggested that tumours with high MCT1 and low MCT4 expression might derive greater benefit, underscoring the critical need for predictive biomarker‐driven patient selection. Because most metabolic‐inhibitor trials have enrolled patients with non‐GC malignancies or mixed solid tumours, we explicitly label each study as direct GC evidence, indirect mixed solid‐tumour evidence or indirect non‐GC evidence. These data should therefore be interpreted as hypothesis‐generating for GC unless GC‐specific cohorts or biomarker‐selected GC subgroups were included. Table 2 provides a biomarker and therapeutic‐strategy evidence hierarchy. Table 3 summarises representative preclinical glycolysis‐targeting agents in GC models, including their proposed mechanisms and reported in vitro and in vivo activities. Table 4 summarises representative clinical studies of metabolic target‐related interventions, with each entry labelled according to GC‐specificity and clinical relevance.^234^, ^235^, ^236^, ^237^, ^238^, ^239^, ^240^, ^241^, ^242^

**TABLE 4:** Clinical studies of metabolic target‐related interventions in cancer.

Metabolic target	Drug	Trial ID	Cancer type	GC‐specificity	Intervention details	Trial results	Translational evidence level	Phase	Status	References
Glycolytic inhibitor	2‐Deoxy‐D‐glucose (2‐DG)	NCT00096707	Lung cancer, breast cancer, pancreatic cancer, head and neck cancer, gastric cancer	Mixed evidence; GC included but no GC‐specific efficacy cohort reported	2‐DG, recommended dose 63 mg/kg/day	Recommended dose of 63 mg/kg/day was identified with tolerable adverse events; clinical benefit was observed in some patients, though corrected Q‐T interval prolongation was noted.	Early clinical mixed‐tumour evidence	I	Completed	^233^
PFKFB3 inhibitor	PFK‐158	NCT02044861	Solid tumours	Indirect mixed solid‐tumour evidence; no GC‐specific cohort reported	Not available	No serious adverse events were reported over a 1‐year observation period, indicating acceptable safety.	Indirect early clinical evidence	I	Unknown	—
PKM2 activator	TP‐1454	NCT04328740	Solid tumours, anal cancer	Indirect non‐GC evidence	Not available	No safety or efficacy data were reported.	Conceptual or early clinical evidence	I	Completed	—
	ACT001	ChiCTR‐OIC‐17013604	Gliomas	Indirect non‐GC evidence	Dose cohorts were used, but exact dose and duration are unavailable.	ACT001 was safe and well tolerated; clinical responses including pathologic responses were observed in a subset of patients at lower dose cohorts.	Indirect early clinical evidence	I	Completed	—
		ChiCTR2000035315	Brain metastases of solid tumours	Indirect non‐GC evidence	ACT001 combined with whole‐brain radiotherapy; exact drug dose and duration are unavailable.	ACT001 combined with whole brain radiotherapy (WBRT) appeared to ameliorate WBRT‐induced adverse events and reduce intracranial tumour burden.	Indirect early clinical evidence	Ib/IIa	Completed	—
PDK inhibitor	Dichloroacetate (DCA)	NCT05120284	Glioblastoma	Indirect non‐GC evidence	Not available	No safety or efficacy data were reported.	Indirect ongoing clinical evidence	II	Active	—
		NCT01111097	Brain tumours	Indirect non‐GC evidence	Oral DCA with GSTZ1/MAAI haplotype‐guided dosing; DLT assessed over 4 weeks; mean treatment duration 75.5 days (range, 26–312) among patients completing ≥1 cycle.	Chronic oral DCA was feasible and well tolerated at doses established for metabolic diseases in patients with recurrent malignant gliomas and brain metastases.	Indirect early clinical evidence	I	Completed	^234^
		NCT01386632	Head and neck squamous cell cancer	Indirect non‐GC evidence	Oral DCA plus cisplatin‐based chemoradiotherapy; dose and duration are not available.	Addition of DCA to cisplatin‐based chemoradiotherapy was safe, with no detrimental effect on survival and expected metabolite changes versus placebo.	Indirect randomised clinical evidence	II	Completed	^235^
		NCT01029925	Advanced non‐small cell lung cancer	Indirect non‐GC evidence	Not available	No clinical benefit was observed in patients with advanced non‐small cell lung cancer and the trial was terminated.	Indirect negative clinical evidence	II	Terminated	^247^
		NCT00566410	Solid tumours	Indirect mixed solid‐tumour evidence	Not available	No safety or efficacy data were reported.	Limited indirect evidence	I	Completed	—
PDK inhibitor, α‐ketoglutarate dehydrogenase inhibitor	Devimistat (CPI‐613)	NCT03793140	Lymphoma, leukaemia	Indirect haematologic malignancy evidence	Not available	Targeting the tricarboxylic acid cycle with devimistat showed limited but clinically meaningful activity in a subset of patients with relapsed/refractory Burkitt lymphoma, a MYC‐driven aggressive B‐cell lymphoma.	Indirect clinical evidence	II	Active	^248^
		NCT03504423	Metastatic pancreatic cancer	Indirect non‐GC evidence	Devimistat 500 mg/m^2^ intravenously on Days 1 and 3, combined with modified FOLFIRINOX on Day 1 of each 14‐day cycle; treatment was continued until disease progression, unacceptable toxicity, withdrawal of consent or other protocol‐defined discontinuation.	Addition of devimistat to modified FOLFIRINOX did not improve outcomes in metastatic pancreatic cancer; no new toxicity signals were observed.	Indirect negative phase III evidence	III	Completed	^249^
		NCT04203160	Biliary tract cancer	Indirect non‐GC evidence	Not available	No safety or efficacy data were reported.	Limited indirect evidence	Ib/II	Completed	—
		NCT03504410	Relapsed/refractory acute myeloid leukaemia (AML)	Indirect haematologic malignancy evidence	Devimistat combined with chemotherapy; dose and duration are not available.	Addition of devimistat to chemotherapy did not improve remission rates or overall survival in relapsed/refractory AML; trial was terminated.	Indirect negative phase III evidence	III	Terminated	^250^
		NCT01835041	Metastatic pancreatic cancer	Indirect non‐GC evidence	Devimistat 500 mg/m^2^ IV on Days 1 and 3 plus modified FOLFIRINOX on Day 1 of each 14‐day cycle; repeated every 2 weeks until progression, unacceptable toxicity or protocol‐defined discontinuation.	The maximum tolerated dose of devimistat was established at 500 mg/m^2^ in combination with modified FOLFIRINOX in metastatic pancreatic cancer.	Indirect early clinical evidence	I	Completed	^236^
		NCT01034475	Haematologic malignancies	Indirect haematologic malignancy evidence	Not available	Devimistat inhibited mitochondrial function and showed activity in heavily pretreated patients with haematologic malignancies.	Indirect early clinical evidence	I	Completed	^237^
		NCT01766219	Biliary tract cancer	Indirect non‐GC evidence	Not available	No safety or efficacy data were reported.	Limited indirect evidence	I/II	Completed	—
		NCT02484391	AML	Indirect haematologic malignancy evidence	Not available	Phase I trial reported feasibility of combining devimistat with cytarabine and mitoxantrone in relapsed/refractory AML.	Indirect early clinical evidence	I	Completed	^238^
Mitochondrial complex I (MCI) inhibitor	Metformin	NCT01101438	Breast cancer	Indirect non‐GC evidence	Metformin was administered orally at 850 mg once daily for the first 4 weeks, then escalated to 850 mg twice daily if tolerated; treatment continued for up to 5 years versus matching placebo.	No significant improvement in invasive disease‐free survival was observed versus placebo in breast cancer.	Indirect negative phase III evidence	III	Completed	^239^, ^240^
		NCT02339168	Prostate cancer	Indirect non‐GC evidence	Not available	Combination with enzalutamide was well tolerated and clinically active in castration‐resistant prostate cancer.		Ib/II	Completed	—
		NCT02325401	Head and neck squamous cell cancer	Indirect non‐GC evidence	Not available	Encouraging overall survival and progression‐free survival were observed when combined with chemoradiotherapy in head and neck squamous cell carcinoma.	Indirect early clinical evidence	I	Completed	^241^
		NCT02362594	Melanoma	Indirect non‐GC evidence	Not available	No significant impact on pembrolizumab efficacy was observed in stage III melanoma.	Indirect clinical evidence	III	Active	^242^
		NCT03800602	Metastatic colorectal cancer	Indirect non‐GC evidence	Metformin was administered as a 14‐day lead‐in monotherapy with gradual dose escalation to 1000 mg orally twice daily. This was followed by nivolumab 480 mg intravenously every 4 weeks in combination with metformin 1000 mg orally twice daily, in 28‐day cycles, for up to 2 years or until disease progression, unacceptable toxicity or withdrawal of consent.	Combination with nivolumab was well tolerated but showed no evidence of efficacy; trends in T‐cell infiltration were noted.	Indirect early clinical evidence	II	Completed	^251^
Mitochondrial inhibitor	ME‐344	NCT02100007	Solid tumours	Indirect mixed solid‐tumour evidence	ME‐344 was administered at 10 mg/kg intravenously once weekly on Days 1, 8, 15 and 22, in combination with topotecan 4 mg/m^2^ on Days 1, 8 and 15, in 28‐day cycles. Treatment cycles were repeated until disease progression or unacceptable toxicity.	Combination with topotecan was tolerable but insufficient anti‐tumour activity to support further investigation.	Indirect early clinical evidence	Ib	Completed	—
		NCT02806817	HER2‐negative breast cancer	Indirect non‐GC evidence	Bevacizumab 15 mg/kg IV single dose on Day 1, followed by ME‐344 10 mg/kg IV over 30 min on Days 8, 15 and 22; surgery was performed on Day 28.	Significant biological anti‐tumour activity was observed as monotherapy in HER2‐negative breast cancer.	Indirect early clinical evidence	I	Completed	—
MCT1 inhibitor	AZD3965	NCT01791595	Solid tumours	Mixed solid‐tumour evidence; no GC‐specific cohort reported	AZD3965 was administered orally as continuous daily dosing in 28‐day cycles; dose escalation evaluated 5–30 mg once daily and 10 or 15 mg twice daily, and the recommended phase II dose was established as 10 mg orally twice daily.	Achieved target engagement by disrupting MCT1‐mediated lactate transport, but showed limited monotherapy anti‐tumour efficacy in unselected advanced cancers.	Early clinical evidence	I	Completed	^44^

Systematic clinical evaluation of these glycolysis‐targeting agents specifically in GC cohorts remains insufficient, representing a significant gap in the translational pipeline. This limitation primarily stems from dose‐limiting systemic toxicities observed with systemic glycolytic blockade, insufficient selectivity for tumour over normal tissues, tumour metabolic plasticity that enables compensatory oxidative phosphorylation, fatty‐acid oxidation or glutamine‐dependent escape under glycolytic stress and the potential impairment of effector T cells function, which also depends on aerobic glycolysis. Bridging this gap therefore requires tumour‐selective delivery systems, pharmacodynamic biomarker monitoring and rational combination therapies designed to anticipate metabolic adaptation rather than simply intensify glycolytic suppression.^44^, ^233^, ^243^, ^244^, ^245^

The negative, terminated or weakly active trials in Table 4 provide several practical lessons. First, unselected monotherapy is unlikely to be sufficient because tumours can bypass glycolytic blockade through oxidative phosphorylation, fatty‐acid oxidation, glutamine utilisation or compensatory lactate transport. Second, systemic glycolytic inhibition may be constrained by narrow therapeutic windows, exemplified by QTc prolongation with 2‐DG and limited efficacy or toxicity‐related discontinuation in several non‐GC trials. Third, the absence of GC‐specific enrichment and pharmacodynamic readouts make it difficult to determine whether target engagement translated into meaningful metabolic remodelling in the tumour. Future trials should therefore prioritise biomarker‐selected GC cohorts, such as MCT1‐high/MCT4‐low, high‐LDH or high‐lactate tumours, FDG‐avid disease or lactylation‐enriched phenotypes and should incorporate longitudinal pharmacodynamic markers, paired tissue or liquid biopsies and rational combinations with chemotherapy, ICIs, anti‐angiogenic therapy or TME‐modulating interventions.^233^, ^246^, ^247^, ^248^, ^249^, ^250^, ^251^

### 4.3 Combination strategies

Given these limitations, the clinical value of Warburg effect targeting is more likely to arise from biomarker‐selected combinations than from unselected monotherapy. Rational strategies should pair metabolic modulation with chemotherapy, ICIs, anti‐angiogenic therapy or TME‐directed interventions according to dominant metabolic dependencies, lactate‐transport status and immune contexture.

#### 4.3.1 With chemotherapy

Metabolic reprogramming is intimately linked to chemoresistance. HK2 contributes to cisplatin resistance through the enhancement of autophagy, and targeting HK2 can restore cisplatin sensitivity.^252^ Similarly, the lesser‐characterised hexokinase family member HKDC1 is up‐regulated in GC and promotes cisplatin and oxaliplatin resistance through a coordinated program of enhanced glycolysis and epithelial–mesenchymal transition; genetic silencing of HKDC1 restores chemosensitivity.^253^ The HSP90–glycolytic enzyme complex is enriched in chemoresistant cells, and the combined inhibition of HSP90 and glycolysis produces synergistic restoration of cisplatin sensitivity.^221^

#### 4.3.2 With immunotherapy

Tumour‐derived lactate is a central mediator of immunosuppression. The PDK inhibitor dichloroacetate (DCA) reprogrammed metabolism towards oxidative phosphorylation, increasing the acetyl‐CoA pool and enhancing H3K27 acetylation at the PD‐L1 promoter. This results in up‐regulated PD‐L1 expression and improved anti‐PD‐L1 efficacy, representing a ‘metabolic enhancement’ strategy that may convert immunologically cold tumours into hot ones.^185^ Additionally, the anthelmintic drug niclosamide suppresses GC glycolysis through a distinct mechanism: it inhibits the m6A reader protein YTHDF2, thereby destabilising LDHA mRNA, reducing lactate production and disrupting the lactate shuttle between tumour cells and endothelial cells within the microenvironment.^254^ Crucially, these metabolic interventions offer a compelling mechanistic rationale for combination regimens by directly reversing or remodelling the acidic, high‐lactate microenvironment detailed in Section 3.3. By dampening lactate accumulation and neutralising extracellular acidity, these inhibitors effectively dismantle the metabolic constraints that exhaust effector CD8‐positive T cells and drive M2 TAMs polarisation. Consequently, concurrently blocking adaptive metabolic circuits while administering ICIs achieves a synergistic effect, successfully shifting the hostile, immune‐evasive TME into an immune‐permissive landscape optimised for robust anti‐tumour surveillance.

#### 4.3.3 With targeted therapy

With targeted therapy in HER2‐positive GC, LDHA is transcriptionally driven by a HER2–HIF‐1α signalling axis, and PFKFB3 inhibition has been shown to improve sensitivity to trastuzumab, partly through normalisation of the tumour vasculature.^255^, ^256^ Nanoparticle‐based delivery platforms offer a means to simultaneously disrupt multiple oncogenic pathways. A solid lipid nanoparticle system co‐delivering miR‐125 and the pan‐tyrosine kinase inhibitor afatinib synergistically suppressed both PI3K/AKT/mTOR signalling and glycolytic enzyme expression (HK2, LDHA), demonstrating potent anti‐tumour activity in preclinical GC models and exemplifying the potential of metabolic–genetic combination therapies.^257^

#### 4.3.4 *H. pylori*‐directed metabolic interventions

In addition to targeting tumour‐intrinsic glycolytic enzymes, the etiological driver of GC—*H. pylori*—represents a clinically actionable axis for combination strategies integrating antimicrobial, metabolic and signalling‐directed interventions. For primary prevention, meta‐analyses have demonstrated that *H. pylori* eradication reduces GC incidence (RR = .61, 95% CI.47–.79).^258^ In the secondary prevention setting, post‐resection eradication significantly decreases metachronous gastric lesions (RR = .54, 95% CI.44–.65).^259^ Aside from conventional eradication, emerging strategies directly intercept oncogenic signals driven by *H. pylori* virulence factors. The CagA oncoprotein enhances glycolytic reprogramming via HIF‐1α up‐regulation and elevates the m^6^A demethylase FTO through Jun‐dependent transcriptional activation.^260^ This ‘hit‐and‐run’ mechanism persists after bacterial clearance, providing a rationale for combining antibiotics with the FTO inhibitor meclofenamic acid (MA), which substantially suppressed CagA‐induced EMT and metastasis in preclinical models.^261^ Mechanistically, the *H. pylori*–NF‐κB–UPP1–glycolysis axis links infection‐driven inflammation to metabolic rewiring; UPP1 up‐regulation enhances glycolytic flux and tumour proliferation, raising the possibility that UPP1 inhibition combined with glycolysis‐targeting agents could block redundant metabolic pathways in *H. pylori*‐associated GC.^262^ Nanoparticle‐based delivery systems have been explored to improve bioavailability and gastric retention of both antibiotics and metabolic inhibitors,^263^ and urease inhibitors constitute another emerging intervention modality.^264^ Collectively, these findings position *H. pylori* as a source of tractable molecular vulnerabilities‐spanning CagA‐driven signalling, FTO‐mediated epitranscriptomics and infection‐induced glycolytic rewiring—that may be therapeutically exploited. Future biomarker‐driven trials stratifying patients by *H. pylori* status, CagA positivity and associated metabolic signatures will be essential to translate these preclinical insights into clinical benefit. These *H. pylori*‐directed strategies are also integrated into Figure 5 as upstream microbiota‐directed interventions that may modulate infection‐driven glycolytic remodelling.

## 5 PERSPECTIVES AND FUTURE DIRECTIONS

### 5.1 Single‐cell metabolomics and spatial resolution

Accumulating evidence underscores that the crosstalk between CAFs and GC cells within the TME is profoundly reshaped by glucose metabolism. Consequently, targeting these metabolic checkpoints emerges as a promising strategy to circumvent immune evasion in GC. However, definitive identification of key interactors and their functional validation within these regulatory networks necessitate further integration of CRISPR–Cas9‐mediated gene editing and comprehensive proteomic profiling.^15^ Furthermore, the functional diversity of CAFs subpopulations remains poorly characterised; thus, refined patient stratification based on CAFs molecular signatures is imperative for maximising the efficacy of targeted interventions. The clinical translation of these metabolic targets requires rigorous preclinical and clinical appraisal, where validation in orthotopic animal models and large‐scale human primary specimens is indispensable. Beyond GC, validating these metabolic nodes in other lactate‐rich malignancies, such as pancreatic and colorectal cancers, may provide a conceptual framework for pan‐cancer therapeutic interventions.^15^, ^216^ Notably, recent observations of PDE5A‐positive CAFs contributing to immune suppression in GC suggest that TME remodelling is susceptible to tumour heterogeneity—including genetic, epigenetic and microenvironmental variables—which dictates distinct stromal interactions and differential responses to PDE5A‐mediated signalling. Moreover, inter‐sample variability (e.g., TNM stage, molecular subtypes or prior treatment history) may limit the generalisability of current findings.^216^ Future longitudinal studies employing single‐cell spatial transcriptomics and multi‐institutional cohorts will be pivotal in elucidating the context‐dependent roles of CAFs and enhancing their prognostic and therapeutic utility. Recent spatial, single‐cell and multi‐omics studies further support the need to move beyond bulk glycolytic markers towards spatially resolved metabolic phenotyping, as metabolic states may differ across tumour nests, stromal regions, immune compartments and metastatic niches.^265^, ^266^, ^267^, ^268^, ^269^, ^270^, ^271^

The metabolic landscape of CAFs is also significantly altered by DGC cells with high metastatic potential. Previous studies have established that exosomal miRNAs and lncRNAs can modulate metabolic phenotypes via extracellular vesicles (EVs)‐mediated delivery; concurrently, TGF‐β and PDGF have been shown to trigger aerobic glycolysis in CAFs by inhibiting IDH3α and enhancing HIF‐1α stability.^272^, ^273^, ^274^ However, in the experimental model presented by Akiko et al., neither alterations in HIF‐1α/HIF‐2α stability nor the involvement of cancer‐derived EVs were detected, implying that alternative humoral factors may govern glucose metabolism in fibroblasts.^217^ Emerging evidence suggests that TNF‐α is capable of orchestrating glucose metabolism in cancer cells independently of HIF‐1α stabilisation.^275^, ^276^ Although the molecular underpinnings of metabolic reprogramming driven by highly metastatic cancer cells in fibroblasts are beginning to surface, the comprehensive machinery governing the metabolic switch from oxidative phosphorylation to aerobic glycolysis remains to be fully elucidated.

### 5.2 Metabolic–immune crosstalk

Previous studies have demonstrated that cells overexpressing SLC2A3 exhibit a high degree of glucose addiction, and enhanced glycolysis drives rapid cell growth and proliferation in vitro. However, the mechanisms by which aberrant glucose metabolism regulates malignant phenotypes and related signalling pathways remain incompletely elucidated.^204^ The mitochondrial topoisomerase TOP1MT, a novel metabolic regulator, has been suggested to attenuate the Warburg effect, normalise glucose metabolic profiles and thereby suppress the progression of certain cancer types.^277^ This may represent a novel strategy for reversing the malignant biological behaviours of tumours, although this hypothesis warrants further in‐depth investigation. The roles of glucose addiction and the Warburg effect in triggering M2 TAMs polarisation and mediating immunosuppression in GC have been extensively documented. Nevertheless, studies integrating spatial metabolomics with single‐cell transcriptomics to dissect the metabolic heterogeneity of TAMs remain scarce.^278^ Pan‐cancer analyses have revealed that the proliferation and cytokine secretion of CD4‐positive helper T cells and effector T cells are highly glycolysis dependent. Consequently, these cells may become functionally inactivated within the TME as a result of glucose deprivation and elevated lactate concentrations.^279^, ^280^ In contrast, energy production in Tregs is less dependent on glycolysis and more reliant on oxidative phosphorylation. This metabolic characteristic enables Tregs to better adapt to low‐glucose and high‐lactate TME while sustaining their immunosuppressive functions.^281^, ^282^ High lactate levels can impair NK cells function by reducing the release of soluble granular components, such as perforin and granzyme, and decreasing the production of cytokines including IFN‐γ and TNF‐α. Additionally, lactate indirectly inhibits NK cells activity by promoting the infiltration of myeloid‐derived suppressor cells.^143^, ^283^ Furthermore, LA is involved in regulating angiogenesis and tissue remodelling, processes that subsequently promote tumour growth and invasion.^284^, ^285^ Although accumulating evidence indicates that immunosuppression induced by an acidic TME may be a key driver of GC progression, the underlying mechanisms by which LA modulates GC progression remain insufficiently elucidated. Moreover, therapeutic strategies aimed at disrupting the highly acidic TME require further validation.

### 5.3 Bridging the preclinical gap: Organoids and humanised in vivo models

Although numerous studies have highlighted the importance and clinical potential of lactate in GC heterogeneity, it is important to note that most experiments are conducted in vitro, which may not adequately reflect the complex interactions and conditions occurring in the body. Therefore, it is necessary to use GC in vitro 3D models or conduct additional in vivo studies to verify the broader significance of lactate in the TME. The microencapsulated SGC7901 cells model, in which cells exhibit normal growth and metabolism and stable protein expression, can form TME similar to those of GC cells, a feature not captured by traditional monolayer cultures. Thus, it provides a relatively stable research platform for further studying the interaction between GC cells and the host microenvironment.^200^ In pan‐cancer studies, it was found that IL‐1β synergistically interacts with IL‐4 to form an acidic TME, which up‐regulates the expression of Fc‐γ receptor IIB in M2 TAMs, thereby inhibiting T cells killing. Although this process has been confirmed in in vitro experiments, more mechanistic studies are needed to explore immunotherapy resistance.^207^ It is important to note that the selection of animal models is crucial in immunotherapy resistance research. Commonly used nude mouse models are difficult to simulate the killing effect of the immune system after activation by PD‐1 antibodies due to the lack of T cells in their immune systems, so mice that are close to the human immune system should be selected as much as possible in the future, rather than the human tumour xenograft models commonly used in animal experiments.^207^, ^286^

### 5.4 Patient stratification and precision monitoring

Early detection of GC and effective monitoring of tumour progression are essential for improving prognosis, reducing the disease burden of GC and lowering its mortality. Current endoscopic and radiological methods are important approaches to enhance the early detection of GC; however, these methods remain invasive, costly and time consuming. Therefore, there is an urgent need to explore new non‐invasive and efficient molecular detection technologies for identifying GC‐related alterations.^287^ Liquid biopsy is a minimally invasive procedure associated with less patient discomfort and a lower risk of complications. It is used to establish the early diagnosis of cancer patients, monitor minimal residual disease and predict prognosis and treatment response, holding potential value for improving personalised medicine in GC patients. However, its low sensitivity and specificity limit its clinical application. Although the combination of multi‐omics information can partially compensate for this deficiency, multi‐omics studies on liquid biopsy samples remain challenging due to the extremely low analyte content in body fluid samples.^288^ Circulating tumour DNA (ctDNA) refers to small genomic fragments released into the circulatory system by tumour cells, which carry gene variants. For patients with a clear pathological diagnosis who cannot undergo further genetic testing due to limited sample collection, ctDNA testing may serve as the preferred non‐invasive tumour screening method, as it can be used to determine the type of genetic mutation.^289^ Metabolic fingerprinting enables rapid, label‐free histopathological diagnosis and prognosis prediction of GC; however, the metabolic characterisation of tissues still faces challenges in terms of analytical tools and sample sources. Teng et al. proposed NPELDI‐MS, a separation‐free mass spectrometry technique that allows rapid analysis with low sample consumption, which holds potential value in assisting clinicopathological diagnosis.^290^

In addition, research on patient stratification also provides guidance for the selection of individualised treatment strategies for GC and the direction of future research. Sheng et al. found that acquired trastuzumab resistance in HER2‐positive GC may be mediated by two distinct mechanisms: induced EMT or endoplasmic reticulum‐associated degradation pathways. Dual‐targeting strategies targeting PD‐1/PD‐L1 and HER2, or HER2 and CLDN18.2, may represent a promising approach to overcome trastuzumab resistance.^265^ Additionally, tumour‐infiltrating lymphocytes (TILs) can be considered as potential predictive markers in GC. Previous studies have demonstrated that PD‐1 blockade may still be effective in patients with elevated CD8‐positive TILs, even in the presence of low PD‐L1 expression.^291^, ^292^, ^293^ The predictive models constructed by Wang et al. may assist in patient stratification to identify personalised treatment strategies.^266^ Through the integration of untargeted metabolomics and machine learning, Zhu et al. identified isovalerylcarnitine (C5) as a key tumour‐suppressive metabolite, which is consistently down‐regulated in both plasma and tissue samples. Isovalerylcarnitine (C5) expression is inversely correlated with the expression of cadherin/MMP family proteins, and it inhibits GC cells migration and invasion, supporting its utility as a potential biomarker and therapeutic target. However, its functional role in the pathogenesis of GC requires further in vivo validation to enhance its potential for clinical translation.^267^ Based on bioinformatics analysis, Liu et al. proposed that the core gene SH3BP1 is a potential tumour suppressor in GC. Its overexpression is associated with lower tumour stage, as well as enhanced CD8‐positive T cells cytotoxicity and infiltration, suggesting that SH3BP1 can serve as a prognostic biomarker and may represent a promising therapeutic target for glucose metabolism reprogramming and immune interactions. Currently, the stability of SH3BP1 as a biomarker and its downstream functions still lack robust experimental verification.^268^

### 5.5 Critical considerations for clinical translation

Several caveats temper the interpretation of Warburg effect‐targeted strategies. First, lactate is not a uniformly immunosuppressive metabolite across all contexts. While lactate accumulation can inhibit cytotoxic T and NK cells function and promote regulatory or myeloid suppressive programs, some immune and stromal subsets may use lactate as a carbon source or respond differently according to oxygen tension, extracellular pH, transporter expression and activation state. Therefore, lactate‐directed therapies should be evaluated with cell‐type‐resolved assays rather than assuming uniform benefit from lactate depletion. Second, systemic inhibition of glycolysis may impair anti‐tumour immunity because activated CD8‐positive T cells and NK cells depend on glycolytic flux for proliferation, cytokine production and cytotoxicity. Thus, agents such as 2‐DG, DCA, LDH inhibitors and MCT inhibitors require careful dose scheduling, tumour‐selective delivery and pharmacodynamic monitoring to avoid suppressing the same immune effector cells that immunotherapy intends to activate. Third, toxicity, selectivity and metabolic compensation remain major barriers. 2‐DG can affect glucose‐dependent normal tissues and has shown cardiac electrophysiologic concerns in early trials; DCA is limited by neuropathy and variable pharmacokinetics; MCT1 blockade may be bypassed by MCT4 expression or oxidative phosphorylation; and single‐agent metabolic inhibitors may select for mitochondrial, fatty‐acid oxidation or glutamine‐dependent escape states. These limitations support biomarker‐selected combinations rather than unselected systemic glycolytic blockade. Accordingly, future trials should prioritise biomarker‐enriched designs, pharmacodynamic monitoring of lactate and glycolytic flux and combination schedules that preserve immune‐cell metabolism while exploiting tumour metabolic vulnerabilities.

## 6 CONCLUSION

In this review, we conceptualise the Warburg effect in GC as an interconnected metabolic ecosystem rather than a simple increase in glycolytic enzyme activity. Unlike previous reviews that have largely catalogued individual regulators, enzymes or inhibitors, our synthesis integrates tumour‐intrinsic signalling, transcriptional and epigenetic regulation, metabolic heterogeneity, lactate‐mediated stromal–immune remodelling, microbiota‐associated metabolic modulation, biomarker development and therapeutic translation into an evidence‐aware framework. We further distinguish direct GC‐derived evidence from indirect evidence extrapolated from other malignancies, underscoring that many proposed metabolic interventions remain preclinical or conceptual. Thus, the Warburg effect represents a biologically compelling but clinically challenging vulnerability in GC. Its successful translation will require robust patient selection, standardised metabolic biomarkers, tumour‐selective delivery strategies, preservation of anti‐tumour immune‐cell metabolism and prospective GC‐specific trials. Future integration of spatial metabolomics, single‐cell analysis, organoid models, microbiome profiling and biomarker‐driven clinical studies may help transform this complex metabolic program into a rational basis for individualised GC therapy. A clinically useful translation of this field will therefore depend less on broad glycolytic suppression than on matching the right metabolic intervention to the right biomarker‐defined patient population.

## AUTHOR CONTRIBUTIONS

Guanmo Liu and Pan Li contributed to conceive, design, revision and oversight of the manuscript sections. Guanmo Liu, Pan Li and Zizhuo Li wrote the manuscript. Jie Li, Chenggang Zhang and Yihua Wang designed the figures and created the tables. Weiming Kang and Xin Ye revised the manuscript. All authors read and approved the final manuscript.

## CONFLICT OF INTEREST STATEMENT

The authors declare no conflicts of interest.

## ETHICS STATEMENT

The authors have nothing to report.

## CONSENT

The authors have nothing to report.

## Data Availability

This manuscript does not report data generation or analysis.
